# Discordant Activity of Kaempferol Towards Dengue Virus and Japanese Encephalitis Virus

**DOI:** 10.3390/molecules25051246

**Published:** 2020-03-10

**Authors:** Chit Care, Wannapa Sornjai, Janejira Jaratsittisin, Atitaya Hitakarun, Nitwara Wikan, Kanokporn Triwitayakorn, Duncan R. Smith

**Affiliations:** Institute of Molecular Biosciences, Mahidol University, Salaya 73170, Thailand; chitcare.cc@gmail.com (C.C.); wannapa.sor@mahidol.ac.th (W.S.); jajanejira@gmail.com (J.J.); tonaor.scmi@gmail.com (A.H.); nitwara.wik@mahidol.ac.th (N.W.); kanokporn.tri@mahidol.ac.th (K.T.)

**Keywords:** Kaempferol, antiviral activity, dengue virus, Japanese encephalitis virus

## Abstract

Kaempferol, a plant-derived flavonoid, has been reported to have activity against Japanese encephalitis virus (JEV) in BHK-21 cells. To determine the broader utility of this compound, we initially evaluated the activity of kaempferol against JEV and dengue virus (DENV) in HEK293T/17 cells. Results showed no significant antiviral activity against either virus. We subsequently investigated the activity of kaempferol against both JEV and DENV in BHK-21 cells. Results showed a significant inhibition of JEV infection but, surprisingly, a significant enhancement of DENV infection. The effect of kaempferol on both host protein expression and transcription was investigated and both transcriptional and translational inhibitory effects were observed, although a more marked effect was observed on host cell protein expression. Markedly, while GRP78 was increased in DENV infected cells treated with kaempferol, it was not increased in JEV infected cells treated with kaempferol. These results show that cellular alteration induced by one compound can have opposite effects on viruses from the same family, suggesting the presence of distinct replication strategies for these two viruses.

## 1. Introduction

The family *Flaviviridae* contains four genera, namely *Flavivirus, Pestivirus, Hepacivirus and Pegivirus* [1]. The genus *Flavivirus* contains the most viral species of the four genera and more than 50 viral species have been assigned to this genus [1]. The *Flavivirus* virion is spherical, approximately 50 nm in diameter and covered by an envelope which surrounds a nucleocapsid with icosahedral symmetry. The genome is a positive sense, single-stranded RNA that consists of a 5’ terminal cap structure, a 5’-untranslated region (UTR), one open reading frame that encodes for three structural proteins (Capsid protein (C), membrane protein (prM/M) and envelope protein (E)) and seven non-structural proteins (NS1, NS2A, NS2B, NS3, NS4A, NS4B, and NS5) and a 3’ UTR without a polyadenylated tail [2,3]. All members of the genus *Flavivirus* are serologically related [4] and are categorized into three clusters based on their main transmission vector (tick-borne, mosquito-borne and no known vector). Mosquito-transmitted flaviviruses such as the four dengue viruses (DENV), Japanese encephalitis virus (JEV), yellow fever virus (YFV) and Zika virus (ZIKV) are the most important arthropod-transmitted human viral pathogens [5].

DENV is the most common arthropod-borne virus and is transmitted by *Aedes* mosquitoes [6]. Every year, approximately 400 million people around the world are infected by DENV [7]. There are four distinct DENV serotypes, DENV 1, DENV 2, DENV 3 and DENV 4 [6], and all serotypes can cause a wide range of illnesses that can be classified as dengue fever (DF), dengue fever with warning signs (DW) or severe dengue (SD) based on the proposed classification of the World Health Organization [8]. While there is a commercially available vaccine for dengue, the utility of this vaccine has been questioned due to an increased risk of severe disease in individuals who were *Flavivirus* naive at the time of vaccination [9].

Japanese encephalitis virus (JEV) is another important mosquito-transmitted *Flavivirus* that is transmitted by *Culex* spp. mosquito [10]. It is associated with considerable mortality and morbidity with approximately 67,900 cases of Japanese encephalitis occurring globally each year, of which 20–30% are fatal and 30–50% of the survivors have significant long-term neurological complications [11]. Although there are several highly effective JEV vaccines available [12], cases of Japanese encephalitis still occur, even in countries such as Thailand [13] in which JEV vaccination is part of the routine childhood vaccination schedule [14]. As noted previously, there are more than 50 viral species in the genus *Flavivirus* and more than half of them are mosquito transmitted viruses associated with human disease [15]. The development of vaccines for each of these viruses is unlikely and thus development of antivirals to treat infections is still a pressing need, and particularly for antivirals that show broad activity against a range of *Flaviviruses*.

There are a number of plant-derived compounds with potential antiviral activity. These include kaempferol (3,4′,5,7-tetrahydroxyflavone, C_15_H_10_O_6_, Supplementary Materials Figure S1) which is a plant-derived flavonoid that can naturally occur in a variety of fruits, vegetables, tea, soy beans and medicinal herbs such as *Coccinia grandis* (ivy gourd; “tamlueng” in Thai) and plants, e.g., *Moringa oleifera* (drumstick tree or horseradish tree; “marum” in Thai) [16]. These medicinal herbs and plants are commonly used to treat a number of health conditions including fever and infection [17,18]. Kaempferol has been reported to exhibit antiviral activity against influenza A virus (H1N1 and H9N2) [19] and human immunodeficiency virus (HIV) 1 [20] under in vitro conditions, and against enterovirus 71 both in vitro and in a mouse model system [21]. It has previously been reported that kaempferol possesses activity against JEV and that kaempferol can inhibit JEV E protein expression as well as expression of the viral mRNA genome [22], but to date there has been no investigation of the potential activity of this compound against other viruses of the genus *Flavivirus*. For this reason, this study aimed to investigate the possible activity of kaempferol against DENV. Initially, kaempferol was shown to possess no activity against either DENV or JEV when the assay was undertaken in HEK293T/17 cells. To explore this, the compound was re-assayed in BHK-21 cells, the cell line which was originally used to show activity against JEV [22]. Remarkably, while kaempferol was able to significantly inhibit JEV infection, it significantly enhanced DENV infection, pointing to the two viruses using distinct mechanisms for their replication.

## 2. Results

### 2.1. Evaluation of the Cytotoxicity of Kaempferol

**Figure 1:**
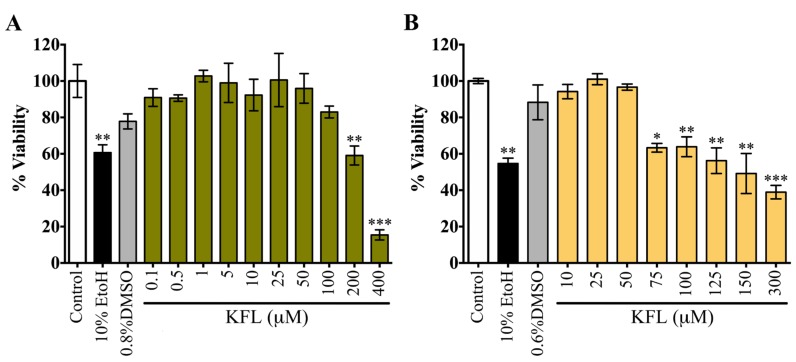
Cytotoxicity of kaempferol. The cytotoxicity of kaempferol was evaluated by using MTT assay. Results are presented as percentage of cell viability from four replicates at 24 h post treatment. (**A**) HEK293T/17 cells and (**B**) BHK-21 cells were treated with various concentrations of kaempferol in parallel with the corresponding percentage of DMSO. Negative (10% FBS in DMEM) and positive (10% EtOH) controls were included. Error bars represent mean ± SD *; *p* value < 0.05, **; *p* value < 0.01 and ***; *p* value < 0.001. All statistics were determined by comparison with the DMSO control.

As part of a study into the potential antiviral activity of kaempferol, we evaluated the cytotoxicity of kaempferol in two cell lines, HEK293T/17 and BHK-21. Both cell lines showed a dose dependent reduction of cell viability compared with the vehicle controls. The CC_50_ of kaempferol for HEK293T/17 cells was 228.5 µM, while the corresponding value for BHK-21 cells was 139.7 µM (Figure 1). In addition, treated cells and controls were evaluated for changes in cell morphology. Obvious morphological changes of HEK293T/17 cells were observed at as low as 25 µM (Supplementary Materials Figure S2), while for BHK-21 cells clear morphological changes were only detected at treatment concentrations of 200 µM and above (Supplementary Materials Figure S3). The corresponding vehicle controls showed no obvious morphological changes in either cell line (Supplementary Materials Figures S2 and S3). In addition, cytotoxicity in BHK-21 cells was assessed by trypan blue staining (Supplementary Materials Figure S4), which showed no detectable cytotoxicity with treatment up to 300 μM. Given this result, we determined the cellular proliferation by counting the number of cells, and a significant loss of cell number was observed. The CC_50_ for kaempferol using this methodology was 168.37 μM, a figure in close agreement with the value determined by the MTT assay. Combined, these results suggest that treatment with high concentrations of kaempferol results in reduced cell proliferation, rather than overt cell death.

### 2.2. Evaluation of Virucidal Activity of Kaempferol

**Figure 2:**
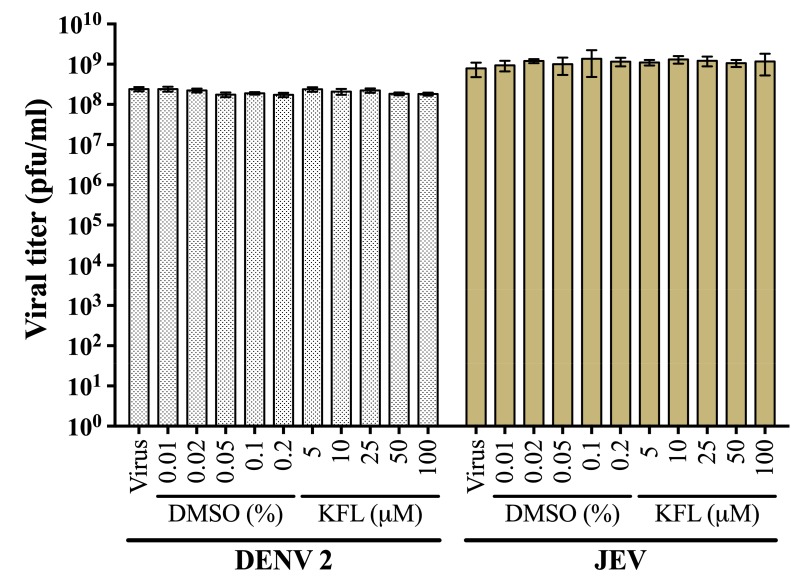
Analysis of direct virucidal activity against dengue virus 2 (DENV 2) and Japanese encephalitis virus (JEV). Either DENV 2 or JEV stock virus were directly incubated with various concentrations of kaempferol or corresponding concentrations of DMSO at 37 °C for 1 h. Subsequently, viral titer in pfu/ml was determined by standard plaque assay. Experiment was performed independently in triplicate with duplicate plaque assay. Error bar shows mean ± SD.

To determine if kaempferol had a directly virucidal activity towards the two viruses, stock virus was incubated with different concentrations of kaempferol or vehicle control for 1 h, before determination of titer by standard plaque assay. Results (Figure 2) showed no significant reduction of viral titer for both viruses after either incubation with kaempferol or vehicle control, suggesting that kaempferol has no direct virucidal activity.

### 2.3. Effects of Kaempferol on DENV 2 and JEV in HEK293T/17 Cells

**Figure 3:**
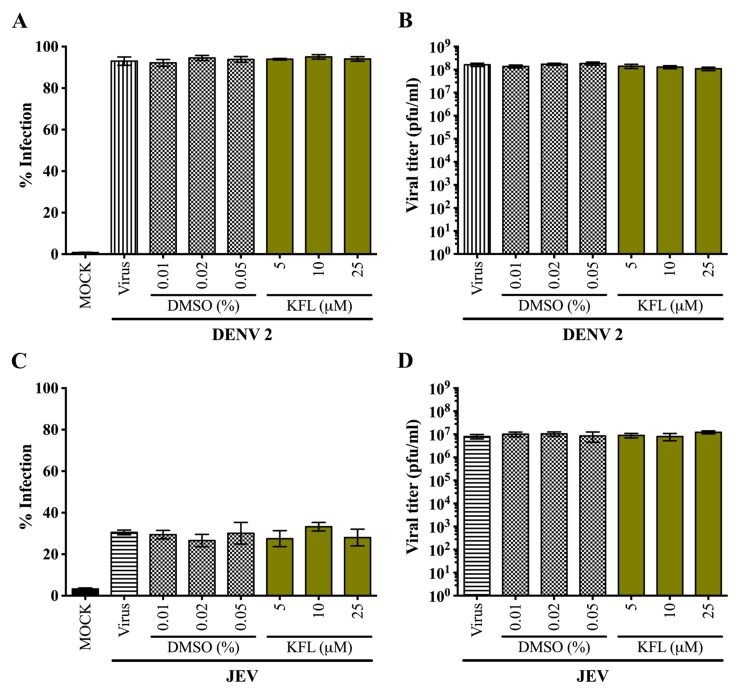
Screening of anti-viral activity of kaempferol against DENV 2 and JEV in HEK293T/17 cells. HEK293T/17 cells were infected with either DENV 2 at MOI 5 or JEV at MOI 2. The infected cells were incubated in the presence or absence of various concentrations of kaempferol or parallel concentrations of DMSO vehicle for 24 h. The percentage infection of DENV 2 (**A**) and JEV (**C**) were analysed by flow cytometry. The viral titer in the supernatants of DENV 2 (**B**) and JEV (**D**) were determined by standard plaque assay. Experiments were undertaken independently in triplicate with duplicate standard plaque assay. Error bars show mean ± SD.

To determine the effects of kaempferol on DENV 2 and JEV infection, HEK293T/17 cells were infected by either DENV 2 (MOI 5) or JEV (MOI 2). After 2 h of infection, the media with the unabsorbed viruses was removed and cells were treated with various concentrations of kaempferol (5, 10 and 25 µM) for 24 h in parallel with cells treated with the corresponding vehicle controls. After 24 h, the cells were evaluated by flow cytometry to determine the percentage infection, while the supernatant was evaluated by plaque assay to determine virus production. Results (Figure 3) showed that treatment with kaempferol had no effect on either DENV or JEV replication.

### 2.4. Effects of Kaempferol on DENV 2 and JEV in BHK-21 Cells

**Figure 4:**
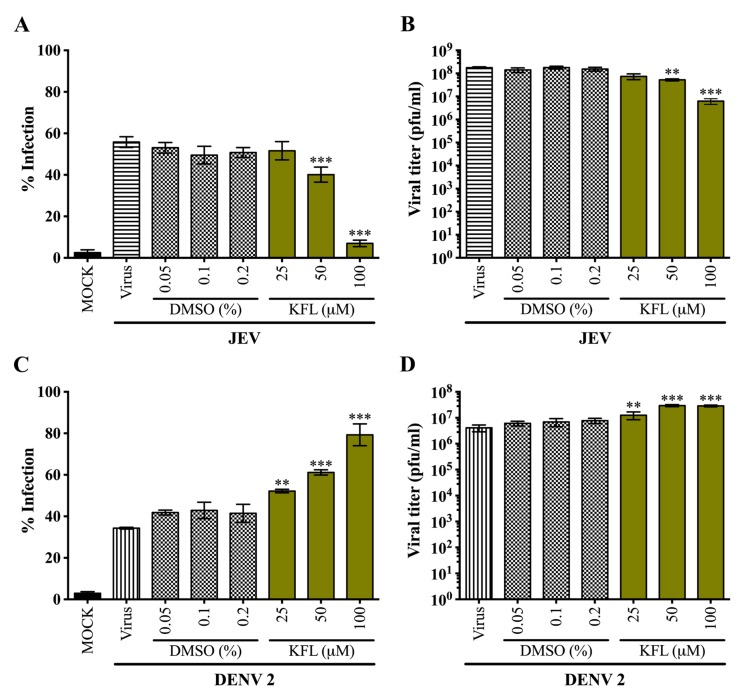
Screening of anti-viral activity of kaempferol against DENV 2 and JEV in BHK-21 cells. BHK-21 cells were infected with either DENV 2 at MOI 5 or JEV at MOI 2. The infected cells were incubated in the presence or absence of various concentrations of kaempferol or parallel concentrations of DMSO vehicle for 24 h. The percentage infection of JEV (**A**) and DENV (**C**) was determined by flow cytometry. The viral production in the supernatants of JEV (**B**) and DENV (**D**) were determined by standard plaque assay. Experiments were undertaken independently in triplicate with duplicate standard plaque assay. Error bars show mean ± SD. **; *p* value ≤ 0.01, ***; *p* value ≤ 0.001. All statistics were determined by comparison with the DMSO control.

The lack of antiviral activity of kaempferol against either virus (DENV or JEV) was somewhat surprising, given the previous reports of activity against JEV [22]. We therefore repeated the experiments, this time using BHK-21 cells. However, given the greater tolerance of BHK-21 cells to kaempferol (based on morphological characteristics) as established above, higher concentrations of drug treatment were employed. Results (Figure 4) for JEV were consistent with the previous reports [22], and a significant reduction in both infection levels and virus production as a result of treatment with kaempferol was observed (Figure 4A,B). The EC_50_ for kaempferol on JEV virus production was 66.33 μM. Surprisingly however, a significant increase in both level of infection and virus production as a consequence of treatment with kaempferol was observed for DENV infection (Figure 4C,D).

### 2.5. Immunofluorescence Assay Analysis of Kaempferol on Infections

**Figure 5:**
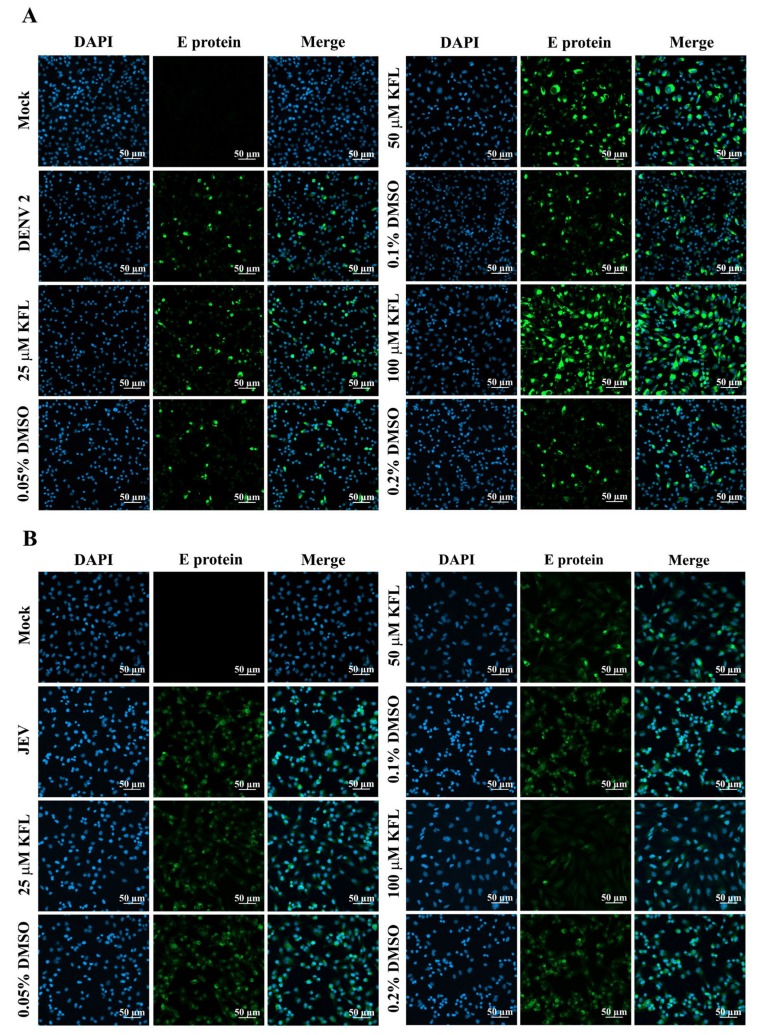
Evaluation of effects of kaempferol by indirect immunofluorescence assay. BHK-21 cells were infected with DENV 2 (**A**) or JEV (**B**). The infected cells were incubated with various concentrations of DMSO or kaempferol for 24 h, after which cells were probed with mouse anti-dengue complex antibody (A) or with a mouse pan specific anti-flavivirus E protein monoclonal antibody (HB112) (**B**). Cells were counterstained with DAPI before observation under a confocal microscope.

To confirm the previous observation of kaempferol inhibiting JEV infection but enhancing DENV infection, BHK-21 cells were again infected with both viruses in the presence or absence of kaempferol and after 24 h the cells were observed under a confocal microscope after staining for E protein expression. As expected, the signal for E protein was markedly reduced in cells infected with JEV and treated with kaempferol, while the signal was markedly enhanced in cells infected with DENV and treated with kaempferol (Figure 5). As such, these results were strongly correlated with the results obtained by flow cytometry and plaque assay.

### 2.6. Effect of Kaempferol on viral Protein Expression

**Figure 6:**
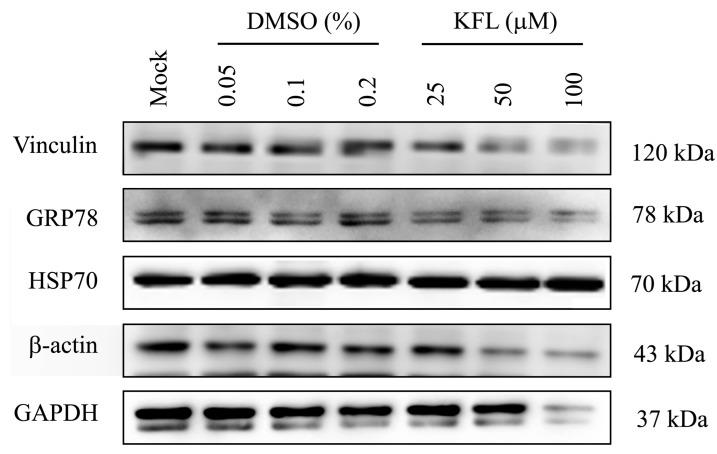
Evaluation of protein expression after kaempferol treatment. BHK-21 cells were treated with different concentrations of kaempferol or DMSO vehicle control for 24 h after which expression of vinculin, GRP78, Hsp70, β-actin and GAPDH was determined by western blotting using appropriate antibodies.

**Figure 7:**
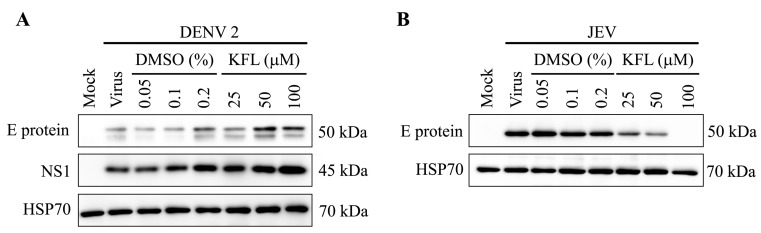
Evaluation of viral protein expression after treatment with kaempferol. BHK-21 cells were mock infected or infected with either DENV 2 at MOI 5 or JEV at MOI 2 followed by 24 h treatment with kaempferol at the indicated concentrations or with corresponding concentrations of DMSO vehicle. The cells were collected at 24 h post treatment and total proteins extracted. Western blot analysis was performed to validate the protein expression of E protein and NS1 in DENV 2 infection (**A**) and JEV E protein in JEV infection (**B**). Hsp70 was used as an internal protein loading control. Experiments were undertaken independently in triplicate.

To further confirm the discordant effect of kaempferol on JEV and DENV infection, viral protein expression after kaempferol treatment was investigated. However, preliminary experiments showed that protein expression for three commonly used protein loading controls (GAPDH, β-actin and vinculin) was markedly down-regulated by kaempferol treatment (Figure 6). We additionally assessed two further proteins, GRP78 and Hsp70. Results (Figure 6) showed that while GRP78 expression was somewhat reduced, expression of Hsp70 was not affected by kaempferol treatment, and this was therefore used as a loading control. BHK-21 cells were therefore mock infected with DENV 2 or JEV and not treated or treated with kaempferol or vehicle control for 24 h.p.i. and the expression of the E protein of both viruses examined by western blotting. Consistent with the previous experiment, DENV E protein expression was increased in response to kaempferol treatment, while JEV E protein expression was reduced (Figure 7).

### 2.7. Effect of Kaempferol on Gene Expression

**Figure 8:**
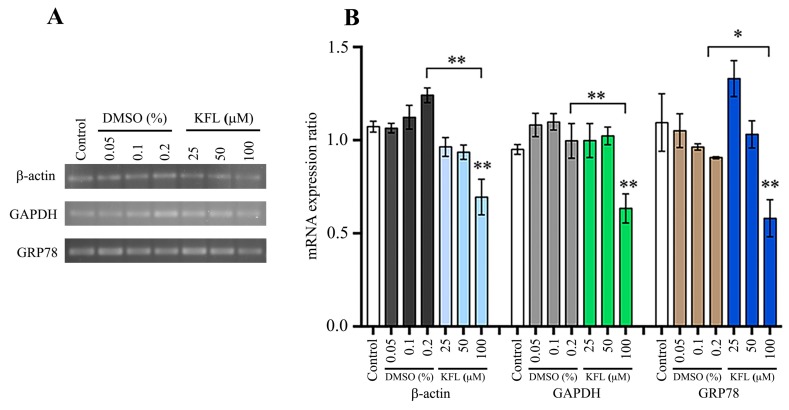
Effect of kaempferol treatment on gene transcription in BHK-21 cells. BHK-21 cells were treated with different concentrations of kaempferol or DMSO vehicle control for 24 h after which expression of β-actin, GAPDH and GRP78 was determined by semi-quantitative PCR (**A**). The band intensity was quantitated using ImageJ analysis software and analysed by GraphPad Prism7 program. The relative expression level of all transcripts was quantitated against the average value and the analysis is presented as bar graph (**B**). Experiment was undertaken independently in triplicate and data was normalized against the average signal for all conditions. Error bars represent SD, *; *p* value ≤ 0.05, **; *p* value ≤ 0.01. All statistics were evaluated by comparing between the treated samples and the equivalent DMSO control.

**Figure 9:**
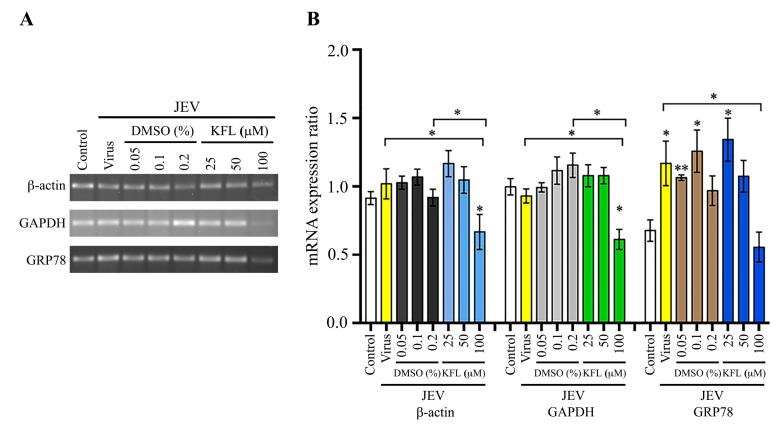
Effect of kaempferol treatment on gene transcription of BHK-21 cells infected with JEV. BHK-21 cells were infected with JEV at MOI 2 and subsequently incubated with varying concentrations of kaempferol or vehicle control for 24 h after which expression of β-actin, GAPDH and GRP78 was determined by semi-quantitative PCR (**A**). The band intensity was quantitated using ImageJ analysis software and analysed by GraphPad Prism7 program. The relative expression level of all transcripts was quantitated against the average value and the analysis is presented as bar graph (**B**). Experiment was undertaken independently in triplicate and data was normalized against the average signal for all conditions. Error bars represent SD, *; *p* value ≤ 0.05, **; *p* value ≤ 0.01. Statistical significance was derived from comparison with the control or with the corresponding DMSO control where indicated.

**Figure 10:**
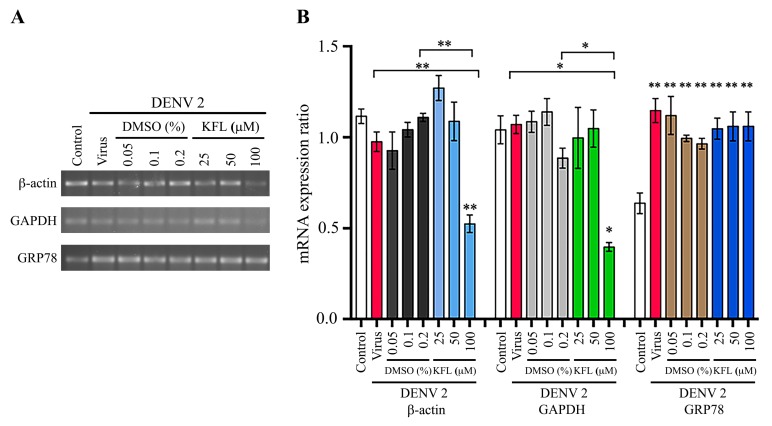
Effect of kaempferol treatment on gene transcription of BHK-21 cells infected with DENV 2. BHK-21 cells were infected with DENV 2 at MOI 5 and subsequently incubated with varying concentrations of kaempferol or vehicle control for 24 h after which expression of β-actin, GAPDH and GRP78 was determined by semi-quantitative PCR (**A**). The band intensity was quantitated using ImageJ analysis software and analysed by GraphPad Prism7 program. The relative expression level of all transcripts was quantitated against the average value and the analysis is presented as bar graph (**B**). Experiment was undertaken independently in triplicate and data was normalized against the average signal for all conditions. Error bars represent SD, *; *p* value ≤ 0.05, **; *p* value ≤ 0.01. Statistical significance was derived from comparison with DENV infection or the DMSO control where indicated.

To further understand the mechanism of action of kaempferol, BHK-21 cells were treated with kaempferol for 24 h, and the level of expression of three genes (β-actin, GAPDH and GRP78) examined by semi-quantitative PCR. The results (Figure 8) showed that the expression of all three genes was significantly reduced by treatment with 100 μM kaempferol. Interestingly, expression of GRP78 was significantly increased at exposure to 25 μM kaempferol (Figure 8). We next determined the expression of the same three genes, but under conditions of infection and treatment with kaempferol. For JEV, while the level of expression of β-actin and GAPDH was approximately equal between control and infected cells with no treatment, the level of GRP78 was significantly increased in response to JEV infection, consistent with previous studies (Figure 9). However, expression of all three genes was reduced in the infected cells treated with 100 μM kaempferol as compared to JEV infected only cells (with no kaempferol treatment). In the DENV infection experiments, results were similar to JEV for β-actin and GAPDH in that levels of expression in control and DENV infected cells with no kaempferol treatment were not significantly different, and levels of expression for both genes was significantly reduced in infected cells treated with 100 μM kaempferol as compared with untreated DENV infected cells (Figure 10). Markedly, however, the results for GRP78 were different from JEV. In particular while expression of GRP78 was increased in untreated DENV infected cells as compared to control cells, the level of expression of GRP78 remained significantly increased in DENV infected cells treated with 100 μM kaempferol (Figure 10).

**Figure 11:**
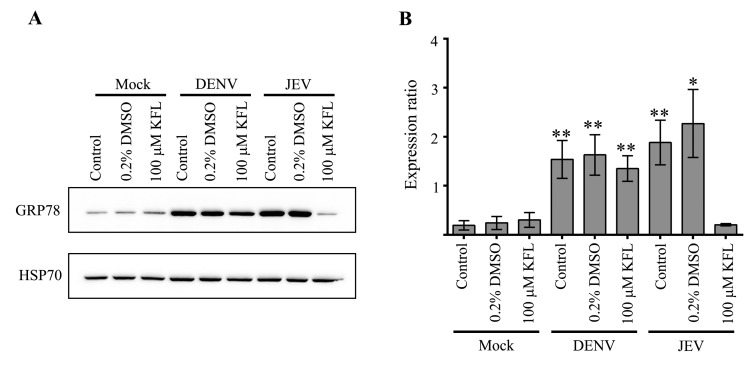
Effect of kaempferol treatment on GRP78 expression. BHK-21 cells were mock infected or infected with JEV or DENV 2 and treated with 100 μM kaempferol, DMSO vehicle or not treated (control) for 24 h. The cells were collected at 24 h post treatment and total proteins were extracted. Western blot analysis was performed to determine the expression of GRP78. Hsp70 was used as an internal protein loading control. Experiments were undertaken independently in triplicate. Error bars represent SD. *; *p* value ≤ 0.05, **; *p* value ≤ 0.01.

Given the maintenance of expression of GRP78 at the transcriptional level in DENV infected kaempferol treated cells, but not in JEV infected and kaempferol treated cells, we examined the expression of GRP78 at the level of the protein. Results (Figure 11) clearly showed that GRP78 protein was increased in DENV infected kaempferol treated cells as compared to mock infected kaempferol treated cells, while GRP78 protein expression was not increased in JEV infected kaempferol treated cells as compared with mock infected, kaempferol treated cells.

## 3. Discussion

Some of the most widespread human pathogenic mosquito transmitted viruses are members of the genus *Flavivirus* such as DENV, YF, JEV and ZIKV. These viruses impose a significant public health burden in many tropical and sub-tropical countries. While there are good commercially available vaccines for YF [23] and JEV [12], the recently introduced commercial vaccine for dengue has not met with great success [9]. For the remaining mosquito transmitted viruses of the genus *Flavivirus*, vaccines are a long way off [24]. Coupled with the lack of protective vaccines for most members of the genus *Flavivirus*, there are no specific antiviral treatments available to directly combat infection. In particular, a drug that is able to treat infections from several members of the genus would be preferred over a specific treatment for one virus.

It is estimated that 80% of the world’s population rely on herbal or medicinal plants to address certain health issues [25]. However, despite this there has been little rigorous analysis in delineating the safety and efficacy of these remedies [25]. While analysis of crudely defined plant extracts is one route to develop antivirals, the evaluation of purified compounds with potential antiviral activity is a route that can be subjected to more rigorous analysis.

Kaempferol is a plant-derived flavonoid that has previously been shown to have antiviral activity against a number of viruses, including JEV [19,20,21,22,26]. This study originally sought to determine whether kaempferol had activity against DENV in the human origin cell line HEK293T/17, a cell line commonly used in studies investigating Flaviviruses, and used JEV as a control. Surprisingly however, no antiviral activity against either virus was seen in this cell line. Repeating the analysis in BHK-21 cells, the cell line originally used to show antiviral activity against JEV [22] showed that kaempferol showed significant antiviral activity against JEV, but enhanced infection by DENV. The reason for the cell type specificity of the activity of kaempferol remains unclear. However, while the 50% cytotoxic concentration (CC_50_) value of kaempferol on HEK293T/17 cells and BHK-21 cells was of a similar order of magnitude (225.8 µM and 139.7 µM, for HEK293T/17 and BHK-21, respectively), morphological changes were seen in HEK293T/17 cells at concentrations as low as 25 μM, while morphological changes were seen in BHK-21 cells only at 200μM or above. For this reason, kaempferol was not investigated as an antiviral agent at levels above 25 μM, while BHK-21 cells were investigated at 100 μM. While it would have been interesting to determine the effects in HEK293T/17 cells at higher concentrations, the obvious morphological changes suggests that this analysis would be of dubious merit. The CC_50_ value for kaempferol determined in BHK-21 cells here (139.7 µM) was slightly lower than that determined previously by Zhang and colleagues (230 µM) [22]. However, Zhang and colleagues [22] determined the CC_50_ value on confluent monolayers, while this study established the CC_50_ on 80% confluent cells, and as such the difference is not unexpected. Similarly, the EC_50_ value determined here (66.33 μM) was somewhat higher than that determined by Zhang and colleagues [22] who reported an EC_50_ of 12.6 μM. Again it is likely that technical differences account for the slightly different values determined.

Markedly, the most surprising result observed in this study was the discordant effect found towards JEV and DENV replication seen in BHK-21 cells. Consistent with previous reports [22], kaempferol exerted a significant antiviral effect on JEV infection, but kaempferol was pro-viral in DENV infection. While attempting to analyze the effect of kaempferol on viral protein expression, we noted that several proteins evaluated for use as a potential loading control (GAPDH, β-actin and vinculin) were significantly reduced by kaempferol treatment. We further evaluated the expression of two additional proteins, namely Hsp70 and GRP78. Hsp70 expression was shown not to be affected by kaempferol, and was therefore used as a loading control. Markedly, GRP78 and Hsp70 were selected for evaluation as the messages for these two proteins have internal ribosome entry site (IRES) sequences [27]. IRES sequences allow cap-independent translation under certain conditions, including cellular stress (reviewed in [28]). IRES sequences were originally identified in viruses [29,30], and since then a number of viruses including DENV and ZIKV have been shown to have functional IRES sequences [31]. However, to date we have been unable to find reports of a functional IRES sequence in JEV. Thus, a compound affecting IRES mediated translation could induce a discordant effect on viral translation. Interestingly, a previous study has shown that kaempferol inhibits enterovirus 71 (EV71) replication by inhibiting IRES mediated translation [32]. However IRES mediated translation is required for EV71 replication [33], while in DENV it is one possible mechanism of translation. More importantly it was shown that kaempferol reduced EV71 IRES mediated translation to approximately 40% [32]. Thus, with DENV even a poorly functioning IRES could enhance replication in the absence of normal cap-mediated translation as compared to normal infection where cap-mediated translation of normal cellular mRNAs could act to limit DENV translation. Zhang and colleagues [22] suggested that kaempferol may exert its anti-JEV activity through directly binding to the JEV RNA. While this is a possible mechanism for the anti-JEV activity, it is unclear how such a binding would lead to an enhancement of infection as seen with DENV. Indeed, if RNA binding was the primary mechanism of inhibition of JEV, it would be likely that the effect on DENV would either be neutral (no effect) or similarly negative.

The effect of kaempferol was examined at the level of transcription both with and without concomitant infection. An inhibition of transcription was seen for all three genes examined (GAPDH, β-actin and GRP78) at the highest concentration examined (100 μM), although some increase in transcription of GRP78 was observed at lower levels. This result suggests that kaempferol does not only exert its effect through translation. Under infection conditions β-actin and GAPDH showed similar reductions in transcription when treated with kaempferol, but, markedly, GRP78 was reduced in JEV infection, though the transcriptional level was maintained in DENV infection. Similarly, at the level of the protein, GRP78 was up-regulated in DENV infected, kaempferol treated cells, but was not up-regulated in JEV infected kaempferol treated cells. GRP78 is critically required for both DENV [34] and JEV [35] infection, and is up-regulated in response to infection by both viruses [34,35]. Previous studies have suggested that kaempferol can affect GRP78 expression, but while some studies suggest that kaempferol up-regulates expression [36,37], other studies have suggested kaempferol down-regulates expression [38,39]. However, while kaempferol could be exerting its effect through GRP78, it is difficult to envision how this would result in discordant results for JEV and DENV infection. More importantly, the results of the proliferation assay support the proposal that kaempferol acts through a shutdown of global translation. In this regards, application of kaempferol as an antiviral may have limited utility in a medical setting.

Several studies have shown that the chaperone protein GRP78 plays a critical role in both DENV [34,40,41,42] and JEV infection [35,43,44,45]. GRP78 is the master regulator of the unfolded proteins response (UPR), a response that is activated during cellular stress [46]. Under non-stressed conditions, GRP78 in the ER sequesters three proteins: inositol-requiring protein 1 (IRE1), activating transcription factor 6 (ATF6) and protein kinase RNA-like endoplasmic reticulum kinase (PERK); while under stressed conditions, GRP78 releases these three proteins which can become activated. Activation of both IRE1 and ATF6 results in up-regulation of GRP78 [47,48,49]. After activation, PERK phosphorylates the alpha subunit of the eukaryotic translation initiation factor 2 (eIF2α) leading to its inactivation, which results in a shutdown of global protein translation [50]. However, eIF2α inhibition does not affect IRES mediated translation. Thus, our results showing reduced expression of housekeeping genes (GAPDH, β-actin and vinculin), but not the IRES containing Hsp70, would again be consistent with kaempferol exerting an effect on cap-mediated translation.

Although the exact mechanism by which DENV and JEV induce ER stress leading to the activation of the UPR and the up-regulation of GRP78 is not known, it is generally believed that it is the influx of viral proteins to the ER that trigger the UPR. Our results are therefore consistent with kaempferol inhibiting cap-dependent translation. This would result in discordant effects on DENV and JEV. DENV, with a characterized IRES [31], could still undergo translation, leading to a viral protein influx to the ER, the triggering of the UPR and the up-regulation of GRP78. The absence of translation from capped messages would enhance the replication of DENV, consistent with the results seen here. In contrast, JEV would not be able to be translated, resulting in no viral influx to the ER, and no up-regulation of GRP78 which is additionally required for JEV replication. Thus, the effect of kaempferol would be antiviral towards JEV, consistent with the results seen here. DENV 2 was utilized in this study as a representative DENV, and it is noted that other DENVs may respond differently. However, overall, the results show that a single drug (kaempferol) can have very discordant effects upon two different members of the genus *Flavivirus*, implicating different replication strategies for these two viruses.

## 4. Materials and Methods

### 4.1. Cells and Viruses

The human embryonic kidney cell line (HEK293T/17) and the baby hamster kidney cell line (BHK-21) were cultured in Dulbecco’s modified Eagle’s medium (DMEM; Gibco, Invitrogen, Waltham, MD) supplemented with 10% (*v*/*v*) heat-inactivated fetal bovine serum (FBS; Gibco, Invitrogen) at 37 °C with 5% CO_2_ in a humidified incubator.

Dengue virus serotype 2 (DENV 2; strain 16681) was propagated in the *Aedes albopictus* derived cell line C6/36 (ATCC No. CRL-1660) exactly as previously described [51]. JEV (Beijing-1 strain) was propagated exactly as described previously [52]. Both viruses were quantitated by standard plaque assay, essentially as previously described [51,52].

### 4.2. Kaempferol

Kaempferol assessed as ≥ 90% purity by high performance liquid chromatography (HPLC) was purchased from Sigma-Aldrich (304401; Sigma-Aldrich, St. Louis, MO). The compound was dissolved with 100% dimethyl sulfoxide (DMSO) to a final stock concentration of 50 mM and stored at −30 °C until required. Various concentrations of kaempferol were prepared by dilution using DMEM with 10% FBS (*v*/*v*).

### 4.3. Cytotoxicity Assays

Either HEK293T/17 cells or BHK-21 were seeded onto 96 well-tissue culture plates at a density that allowed 80% confluence to be reached after 24 h of incubation under standard conditions. The culture medium was removed and then cells were treated with various concentration of DMSO or kaempferol for 24 h following which cytotoxicity was determined by the MTT assay using a MTT Cell Growth Assay Kit (Merck KGaA, Darmstadt, Germany). Cells cultured in complete media or 10% EtOH were used as the negative and positive controls, respectively. Because of interference between the MTT reagent and kaempferol [53], a blank was undertaken (no cells but with kaempferol) and this background reading was subtracted from all experimental readings. The experiment was repeated and this time cells were stained with trypan blue after treatment for 24 h. Live and dead cells were observed under a haemocytometer and viability was determined. In addition, total cell number was determined (proliferation assay).

### 4.4. Effect of Kaempferol on Cell Morphology

Either HEK293T/17 cells or BHK-21 cells were seeded onto 96 well-tissue culture plates at a density that allowed 80% confluence to be reached after 24 h of incubation under standard conditions. These cells were treated with various concentrations of kaempferol or vehicle control. Treated cells were incubated at 37 °C with 5% CO_2_ for 24 h. Cell morphology was observed under an inverted microscope.

### 4.5. Virucidal Assay

DENV 2 or JEV stock virus was incubated directly with various concentrations of kaempferol or vehicle control for 1 h at 37 °C after which the viral titer was determined by standard plaque assay in LLC-MK2 cells (DENV 2) or Vero cells (JEV). Experiment was undertaken independently in triplicate with duplicate plaque assay.

### 4.6. Standard Infection

Either HEK293T/17 cells or BHK-21 cells were seeded onto six well-tissue culture plates at a density that allowed 80% confluence to be reached after 24 h of incubation under standard conditions, after which time media was removed and cells were infected with the appropriate virus at the appropriate MOI in FBS free DMEM for 2 h after which the media was removed and replaced with standard culture media containing kaempferol or vehicle control as appropriate.

### 4.7. Flow Cytometry

Mock or DENV or JEV infected cells (with or without drug treatment) were harvested at 24 h post-infection and then blocked with 10% goat serum (Gibco BRL, Gaitherburg, MD) in PBS on ice for 30 min. The cells were fixed with 200 μL of 4% paraformaldehyde in PBS buffer at room temperature in the dark for 20 min. Cells were subsequently permeabilized with 200 μL of 0.2% Triton X-100 in PBS-immunofluorescence assay (IFA) (150 mM NaCl, 50 mM NaH_2_PO_4_, 50 mM Na_2_HPO_4_, pH to 7.4) for 10 min. DENV infected cells were incubated with 50 µL of a mouse anti-DENV E protein monoclonal antibody (HB114 [54]) at dilution of 1:150, while JEV infected cells were incubated with 50 µL of a mouse pan-specific anti-flavivirus E protein monoclonal antibody (HB112 [54]) at a dilution of 1:2 in 1% BSA/PBS-IFA at 4 °C overnight. The cells were subsequently incubated with 50 µl of a goat anti-mouse IgG polyclonal antibody conjugated with fluorescein isothiocyanate (FITC; KPL, Guilford, UK) at a dilution of 1:40 in 1% BSA/PBS-IFA for 1 h in the dark. Between steps the cells were washed twice with 1 ml of 1% BSA/PBS-IFA. Finally, the cells were resuspended in 200 µL of PBS-IFA and analysed by flow cytometry on a BD FACalibur cytometer (Becton Dickinson, BD Biosciences, San Jose, CA) using CELLQuest™ software. All experiments were undertaken independently in triplicate.

### 4.8. Indirect Immunofluorescence Assay

BHK-21 cells were grown on cover slips in 6-well tissue culture plates for 24 h prior to infection. Then the cells were either mock infected or infected with DENV 2 or JEV for 2 h. The media with unabsorbed viruses was removed and replaced with 10% FBS, DMEM containing vehicle control or kaempferol. The cells were incubated at 37 °C and 5% CO_2_ for 24 h. Infected cells were rinsed thoroughly three times with PBS and the cells were fixed with 4% paraformaldehyde for 20 min at room temperature in the dark. After washing three times with PBS, the cells were permeabilised with 0.3% Triton X-100/PBS-IFA for 10 min and washed twice with 0.03% Triton X-100/PBS-IFA. After permeabilization, the cells were blocked with 10% goat serum (Gibco BRL, Gaitherburg, MD) for 1 h at 4 °C and washed again. Subsequently, the cells were incubated overnight at 4 °C with a mouse anti-dengue complex monoclonal antibody (MAB 8705, Burlington, MA) at a dilution of 1:40 in PBS, or a mouse pan-specific anti-flavivirus E protein monoclonal antibody (HB-112) at a dilution of 1:2 in PBS as appropriate. After washing three times the cells were incubated with 1:40 dilution of donkey anti-mouse IgG polyclonal antibody conjugated with Alexa Fluor 488 (Abcam, Cambridge, UK) and a 1:300 dilution of a DAPI (Molecular Probes, Eugene, OR) in PBS/IFA at room temperature for 1 h in the dark, followed by washing. The cover slips were mounted onto glass slides using Prolong^®^ Gold anti-fade reagent (Invitrogen, Carlsbad, CA). Finally, stained cells were observed and recorded under a confocal microscope (ZEISS LSM 800, Oberkochen, Germany).

### 4.9. Western Blot Analysis

Cells collected by scraping were lysed with 100 µL of radioimmunoprecipitation buffer (RIPA) and kept on ice for 30 min with vortexing every 10 min, and then centrifuged at 12,250× *g* at 4 °C for 15 min. Protein concentrations were measured by Bradford assay [55]. The protein samples were mixed with 5× native dye, boiled for 5 min and centrifuged at 4 °C for 5 min, and then loaded onto discontinuous SDS PAGE gels (5% stacking and 12% separating gel) and run at 120 V constant until the dye ran out from the gel. Then the proteins were transferred onto nitrocellulose membranes. The membranes were blocked with 5% skim milk in TBS containing 0.05% Tween-20 at room temperature for 30 min and subsequently incubated with an appropriate primary antibody overnight at 4 °C, followed by incubation with an appropriate secondary antibody conjugated with HRP for 1 h at room temperature. Antibodies used included a 1:2 dilution of a pan specific anti-flavivirus mouse monoclonal antibody (HB112 [54]), a 1:500 dilution of a mouse anti-dengue virus serotype 1-4 monoclonal antibody (MA1-27093, Thermo Fisher Scientific, Waltham, MA), a 1:5,000 dilution of a mouse anti-GAPDH monoclonal antibody (sc-32333, Santa Cruz Biotechnology Inc., Dallas, TX), a 1:1,000 dilution of a goat anti-GRP78 polyclonal antibody (sc-13968, Santa Cruz Biotechnology Inc.), a 1:1,000 dilution of a goat anti-actin polyclonal antibody (sc-1616, Santa Cruz Biotechnology Inc.), a 1:5,000 dilution of a goat anti-vinculin polyclonal antibody (sc-7649, Santa Cruz Biotechnology Inc.), a 1:2,000 dilution of a rabbit anti-dengue NS1 polyclonal antibody (PAS-27885, Thermo Fisher Scientific) and a 1:2,000 dilution of a rabbit anti-Hsp70 polyclonal antibody (sc-1060-R, Santa Cruz Biotechnology Inc.). Secondary antibodies used were a 1:5,000 dilution of a HRP-conjugated goat anti-mouse IgG polyclonal antibody (A5278, Sigma-Aldrich, St. Louis, MO), a 1:5,000 dilution of a HRP-conjugated goat anti-rabbit IgG polyclonal antibody (31460, Thermo Fisher Scientific) and a 1:5,000 dilution of a HRP-conjugated rabbit anti-goat IgG polyclonal antibody (G4018, Sigma-Aldrich). All experiments were undertaken independently in triplicate.

### 4.10. Semiquantitative PCR

BHK-21 cells were infected with either DENV 2 or JEV, followed by treatment with kaempferol or vehicle control for 24 h, after which total RNA was isolated using Trizol according to the manufacturer’s protocol. RNA concentration was determined using a NanoDrop 2000 spectrophotometer (Thermo Scientific). Then, 1 µg of extracted RNA was used to synthesize cDNA by RT-PCR using RevertAid reverse transcriptase (Thermo Fisher Scientific Inc., Waltham, MA) and oligo (dT) as a primer. Afterward, cDNA was amplified by PCR using gene specific primers (Supplementary Materials Table S1). The amplification products were electrophoresed through 2% agarose gels, stained with ethidium bromide and visualized under UV. The intensity of the gel bands was analysed using ImageJ [56]. Values were internally normalized against the mean of all values.

### 4.11. Statistical Analysis 

The GraphPad Prism program (GrapPad Software Inc., San Diego, CA) was used to create graphs from the raw data. Statistical analysis for significance was undertaken by independent sample T test using SPSS (SPSS Inc., Chicago, IL). CC50 values were calculated using the freeware ED50plus (v1.0) software [57]. 

## Supplementary Materials

The following are available online at https://www.mdpi.com/1420-3049/25/5/1246/s1, Figure S1. Morphological changes of HEK293T/17 cells after treatment with kaempferol. Figure S2. Morphological changes of BHK-21 cells after treatment with kaempferol. Figure S5–S26. Morphological changes of HEK293T/17 cells after treatment with kaempferol. Figure S27–S37. Morphological changes of BHK-21 cells after treatment with kaempferol. Table S1. Primer sequences. File S1. Uncropped western blots.

## References

[1] Simmonds P., Becher P., Bukh J., Gould E.A., Meyers G., Monath T., Muerhoff S., Pletnev A., Rico-Hesse R., Smith D.B. (2017). ICTV virus taxonomy profile: *Flaviviridae*. J. Gen. Virol..

[2] Modis Y., Ogata S., Clements D., Harrison S.C. (2004). Structure of the dengue virus envelope protein after membrane fusion. Nature.

[3] Acheson N.H. (2011). Introduction to Virology.

[4] Calisher C.H., Karabatsos N., Dalrymple J.M., Shope R.E., Porterfield J.S., Westaway E.G., Brandt W.E. (1989). Antigenic relationships between flaviviruses as determined by cross-neutralization tests with polyclonal antisera. J. Gen. Virol..

[5] Gould E.A., Solomon T. (2008). Pathogenic flaviviruses. Lancet.

[6] Gubler D.J. (1998). Dengue and dengue hemorrhagic fever. Clin. Microbiol Rev..

[7] Bhatt S., Gething P.W., Brady O.J., Messina J.P., Farlow A.W., Moyes C.L., Drake J.M., Brownstein J.S., Hoen A.G., Sankoh O. (2013). The global distribution and burden of dengue. Nature.

[8] Hadinegoro S.R. (2012). The revised WHO dengue case classification: does the system need to be modified?. Paediatr Int Child. Health.

[9] Halstead S.B. (2017). Dengvaxia sensitizes seronegatives to vaccine enhanced disease regardless of age. Vaccine.

[10] Solomon T., Dung N.M., Kneen R., Gainsborough M., Vaughn D.W., Khanh V.T. (2000). Japanese encephalitis. J. Neurol. Neurosurg. Psychiatry.

[11] Campbell G.L., Hills S.L., Fischer M., Jacobson J.A., Hoke C.H., Hombach J.M., Marfin A.A., Solomon T., Tsai T.F., Tsu V.D. (2011). Estimated global incidence of Japanese encephalitis: a systematic review. Bull. World Health Organ..

[12] Yun S.I., Lee Y.M. (2014). Japanese encephalitis: the virus and vaccines. Hum. Vaccin Immunother.

[13] Olsen S.J., Supawat K., Campbell A.P., Anantapreecha S., Liamsuwan S., Tunlayadechanont S., Visudtibhan A., Lupthikulthum S., Dhiravibulya K., Viriyavejakul A. (2010). Japanese encephalitis virus remains an important cause of encephalitis in Thailand. Int J. Infect. Dis.

[14] Pongpairoj S., Choakouychai B., Boonrueng C., Kutirakan P., Ahandrik S., Leelasiri K., Phanthumachinda B. (1989). A test production of inactivated mouse brain JE vaccine in Thailand. The Southeast. Asian J. Trop Med. Public Health.

[15] Smith D.R. (2016). Waiting in the wings: The potential of mosquito transmitted flaviviruses to emerge. Crit Rev. Microbiol.

[16] Calderon-Montano J.M., Burgos-Moron E., Perez-Guerrero C., Lopez-Lazaro M. (2011). A review on the dietary flavonoid kaempferol. Mini Rev. Med. Chem.

[17] Verri W.A., Vicentini F.T.M.C., Baracat M.M., Georgetti S.R., Cardoso R.D.R., Cunha T.M., Ferreira S.H., Cunha F.Q., Fonseca M.J.V., Casagrande R. (2012). Flavonoids as anti-inflammatory and analgesic drugs: Mechanisms of action and perspectives in the development of pharmaceutical forms. Studies Nat. Prof. Chem.

[18] Panche A.N., Diwan A.D., Chandra S.R. (2016). Flavonoids: an overview. J. Nutr Sci.

[19] Jeong H.J., Ryu Y.B., Park S.J., Kim J.H., Kwon H.J., Kim J.H., Park K.H., Rho M.C., Lee W.S. (2009). Neuraminidase inhibitory activities of flavonols isolated from *Rhodiola rosea* roots and their in vitro anti-influenza viral activities. Bioorg Med. Chem..

[20] Behbahani M., Sayedipour S., Pourazar A., Shanehsazzadeh M. (2014). In vitro anti-HIV-1 activities of kaempferol and kaempferol-7-Oglucoside isolated from *Securigera securidaca*. Res. Pharm. Sci..

[21] Dai W., Bi J., Li F., Wang S., Huang X., Meng X., Sun B., Wang D., Kong W., Jiang C. (2019). Antiviral efficacy of flavonoids against enterovirus 71 infection *in vitro* and in newborn mice. Viruses.

[22] Zhang T., Wu Z., Du J., Hu Y., Liu L., Yang F., Jin Q. (2012). Anti-Japanese-encephalitis-viral effects of kaempferol and daidzin and their RNA-binding characteristics. PLoS One.

[23] Barrett A.D.T. (2017). Yellow fever live attenuated vaccine: A very successful live attenuated vaccine but still we have problems controlling the disease. Vaccine.

[24] Heinz F.X., Stiasny K. (2012). Flaviviruses and flavivirus vaccines. Vaccine.

[25] Ekor M. (2014). The growing use of herbal medicines: issues relating to adverse reactions and challenges in monitoring safety. Front. Pharmacol..

[26] Mitrocotsa D., Mitaku S., Axarlis S., Harvala C., Malamas M. (2000). Evaluation of the antiviral activity of kaempferol and its glycosides against human cytomegalovirus. Planta Medica.

[27] Mokrejs M., Vopalensky V., Kolenaty O., Masek T., Feketova Z., Sekyrova P., Skaloudova B., Kriz V., Pospisek M. (2006). IRESite: the database of experimentally verified IRES structures (www.iresite.org). Nucleic Acids Res..

[28] Yang Y., Wang Z. (2019). IRES-mediated cap-independent translation, a path leading to hidden proteome. J. Mol. Cell Biol..

[29] Jang S.K., Krausslich H.G., Nicklin M.J., Duke G.M., Palmenberg A.C., Wimmer E. (1988). A segment of the 5’ nontranslated region of encephalomyocarditis virus RNA directs internal entry of ribosomes during in vitro translation. J. Virol.

[30] Pelletier J., Sonenberg N. (1988). Internal initiation of translation of eukaryotic mRNA directed by a sequence derived from poliovirus RNA. Nature.

[31] Song Y., Mugavero J., Stauft C.B., Wimmer E. (2019). Dengue and Zika virus 5’ untranslated regions harbor internal ribosomal entry site functions. mBio.

[32] Tsai F.J., Lin C.W., Lai C.C., Lan Y.C., Lai C.H., Hung C.H., Hsueh K.C., Lin T.H., Chang H.C., Wan L. (2011). Kaempferol inhibits enterovirus 71 replication and internal ribosome entry site (IRES) activity through FUBP and HNRP proteins. Food Chem.

[33] Thompson S.R., Sarnow P. (2003). Enterovirus 71 contains a type I IRES element that functions when eukaryotic initiation factor eIF4G is cleaved. Virology.

[34] Wati S., Soo M.L., Zilm P., Li P., Paton A.W., Burrell C.J., Beard M., Carr J.M. (2009). Dengue virus infection induces upregulation of GRP78, which acts to chaperone viral antigen production. J. Virol..

[35] Nain M., Mukherjee S., Karmakar S.P., Paton A.W., Paton J.C., Abdin M.Z., Basu A., Kalia M., Vrati S. (2017). GRP78 is an important host factor for Japanese encephalitis virus entry and replication in mammalian cells. J. Virol..

[36] Chandrika B.B., Maney S.K., Lekshmi S.U., Joseph J., Seervi M., Praveen K.S., Santhoshkumar T.R. (2010). Bax deficiency mediated drug resistance can be reversed by endoplasmic reticulum stress induced death signaling. Biochem Pharmacol.

[37] Wang H., Chen L., Zhang X., Xu L., Xie B., Shi H., Duan Z., Zhang H., Ren F. (2019). Kaempferol protects mice from d-GalN/LPS-induced acute liver failure by regulating the ER stress-Grp78-CHOP signaling pathway. Biomed. Pharmacother..

[38] Abdullah A., Ravanan P. (2018). Kaempferol mitigates endoplasmic reticulum stress induced cell death by targeting caspase 3/7. Sci Rep..

[39] Kim D.S., Ha K.C., Kwon D.Y., Kim M.S., Kim H.R., Chae S.W., Chae H.J. (2008). Kaempferol protects ischemia/reperfusion-induced cardiac damage through the regulation of endoplasmic reticulum stress. Immunopharmacol. Immunotoxicol..

[40] Chen H.H., Chen C.C., Lin Y.S., Chang P.C., Lu Z.Y., Lin C.F., Chen C.L., Chang C.P. (2017). AR-12 suppresses dengue virus replication by down-regulation of PI3K/AKT and GRP78. Antiviral Res..

[41] Diwaker D., Mishra K.P., Ganju L. (2015). Effect of modulation of unfolded protein response pathway on dengue virus infection. Acta Biochim Biophys Sin. (Shanghai).

[42] Jitobaom K., Tongluan N., Smith D.R. (2016). Involvement of voltage-dependent anion channel (VDAC) in dengue infection. Sci. Rep..

[43] Lyoo H.R., Park S.Y., Kim J.Y., Jeong Y.S. (2015). Constant up-regulation of BiP/GRP78 expression prevents virus-induced apoptosis in BHK-21 cells with Japanese encephalitis virus persistent infection. Virol J..

[44] Mukherjee S., Singh N., Sengupta N., Fatima M., Seth P., Mahadevan A., Shankar S.K., Bhattacharyya A., Basu A. (2017). Japanese encephalitis virus induces human neural stem/progenitor cell death by elevating GRP78, PHB and hnRNPC through ER stress. Cell Death Dis.

[45] Wu Y.P., Chang C.M., Hung C.Y., Tsai M.C., Schuyler S.C., Wang R.Y. (2011). Japanese encephalitis virus co-opts the ER-stress response protein GRP78 for viral infectivity. Virol. J..

[46] Rutkowski D.T., Kaufman R.J. (2004). A trip to the ER: coping with stress. Trends Cell Biol.

[47] Baumeister P., Luo S., Skarnes W.C., Sui G., Seto E., Shi Y., Lee A.S. (2005). Endoplasmic reticulum stress induction of the Grp78/BiP promoter: activating mechanisms mediated by YY1 and its interactive chromatin modifiers. Mol. Cell Biol..

[48] Reddy R.K., Mao C., Baumeister P., Austin R.C., Kaufman R.J., Lee A.S. (2003). Endoplasmic reticulum chaperone protein GRP78 protects cells from apoptosis induced by topoisomerase inhibitors: role of ATP binding site in suppression of caspase-7 activation. J. Biol. Chem..

[49] Scheuner D., Song B., McEwen E., Liu C., Laybutt R., Gillespie P., Saunders T., Bonner-Weir S., Kaufman R.J. (2001). Translational control is required for the unfolded protein response and in vivo glucose homeostasis. Mol. Cell.

[50] Harding H.P., Zhang Y., Bertolotti A., Zeng H., Ron D. (2000). Perk is essential for translational regulation and cell survival during the unfolded protein response. Mol. Cell.

[51] Sithisarn P., Suksanpaisan L., Thepparit C., Smith D.R. (2003). Behavior of the dengue virus in solution. J. Med. Virol..

[52] Boonsanay V., Smith D.R. (2007). Entry into and production of the Japanese encephalitis virus from C6/36 cells. Intervirology.

[53] Bruggisser R., von Daeniken K., Jundt G., Schaffner W., Tullberg-Reinert H. (2002). Interference of plant extracts, phytoestrogens and antioxidants with the MTT tetrazolium assay. Planta Med..

[54] Henchal E.A., Gentry M.K., McCown J.M., Brandt W.E. (1982). Dengue virus-specific and flavivirus group determinants identified with monoclonal antibodies by indirect immunofluorescence. Am. J. Trop Med. Hyg..

[55] Bradford M.M. (1976). A rapid and sensitive method for the quantitation of microgram quantities of protein utilizing the principle of protein-dye binding. Anal. Biochem.

[56] Abramoff M.D., Magelhaes P.J., Ram S.J. (2004). Image processing with Image. J. Biophotonics Int..

[57] Vargas M.H. (2000). ED50plus v1.0. Instituto Nacional de Enfermedades Respiratorias [Computer software]. Mexico, DF, Mexico. http://sciencegateway.org/protocols/cellbio/drug/data/ed50v10.xls.

