# Supplementary material for: Discordant Activity of Kaempferol Towards Dengue Virus and Japanese Encephalitis Virus
Source: Molecules. 2020 Mar 10;25(5):1246. doi: 10.3390/molecules25051246 (PMC7179415; doi:10.3390/molecules25051246)

Supplemental materials

Discordant activity of kaempferol towards dengue virus and Japanese encephalitis virus

Chit Care[1], Wannapa Sornjai[1], Janejira Jaratsittisin[1], Atitaya Hitakarun[1], Nitwara Wikan[1], Kanokporn Triwitayakorn[1], Duncan R. Smith[1]*

[1]Institute of Molecular Biosciences, Mahidol University, Thailand

*Correspondence to

Duncan R. Smith (duncan_r_smith@hotmail.com)

Institute of Molecular Biosciences, Mahidol University, Salaya Campus

25/25 Phuttamontol Sai 4, Salaya, Nakhon Pathom, Thailand 73170

Tel (662) 800 3624-8; Fax (662) 4419906

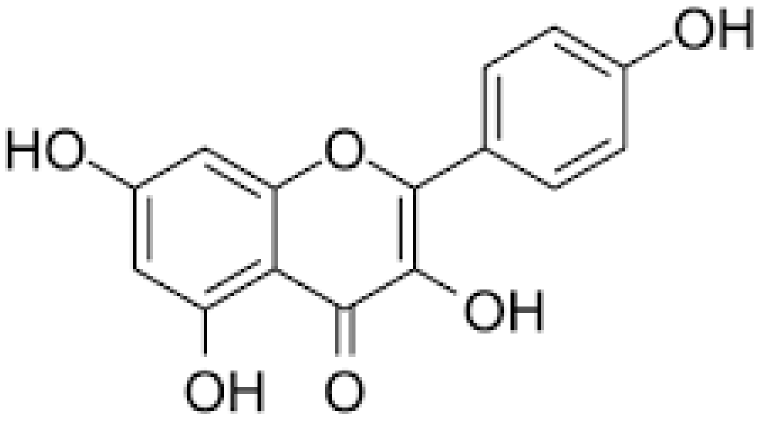

Supplemental Figure S1. Structure of kaempferol.

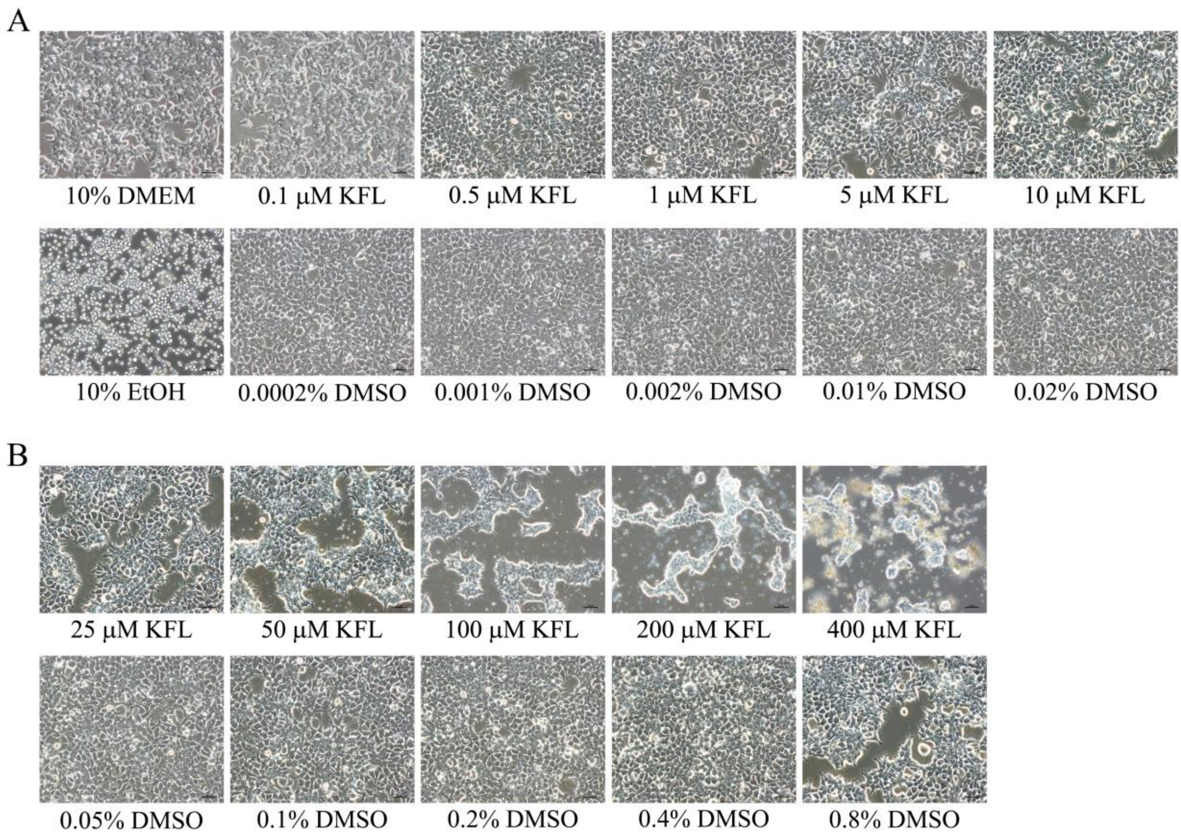

Supplemental Figure S2. Morphological changes of HEK293T/17 cells after treatment with kaempferol

HEK293T/17 cells were treated with various concentrations of DMSO or kaempferol for 24 hrs after which the cell morphology was observed under an inverted microscope. Cells that were treated with 10% EtOH were used as a positive control. All experiments were undertaken independently in duplicate. Magnification: 200 X, Scale bar 20 μm. Enlargements of representative panels can be found in Supplementary Figures S5-26.

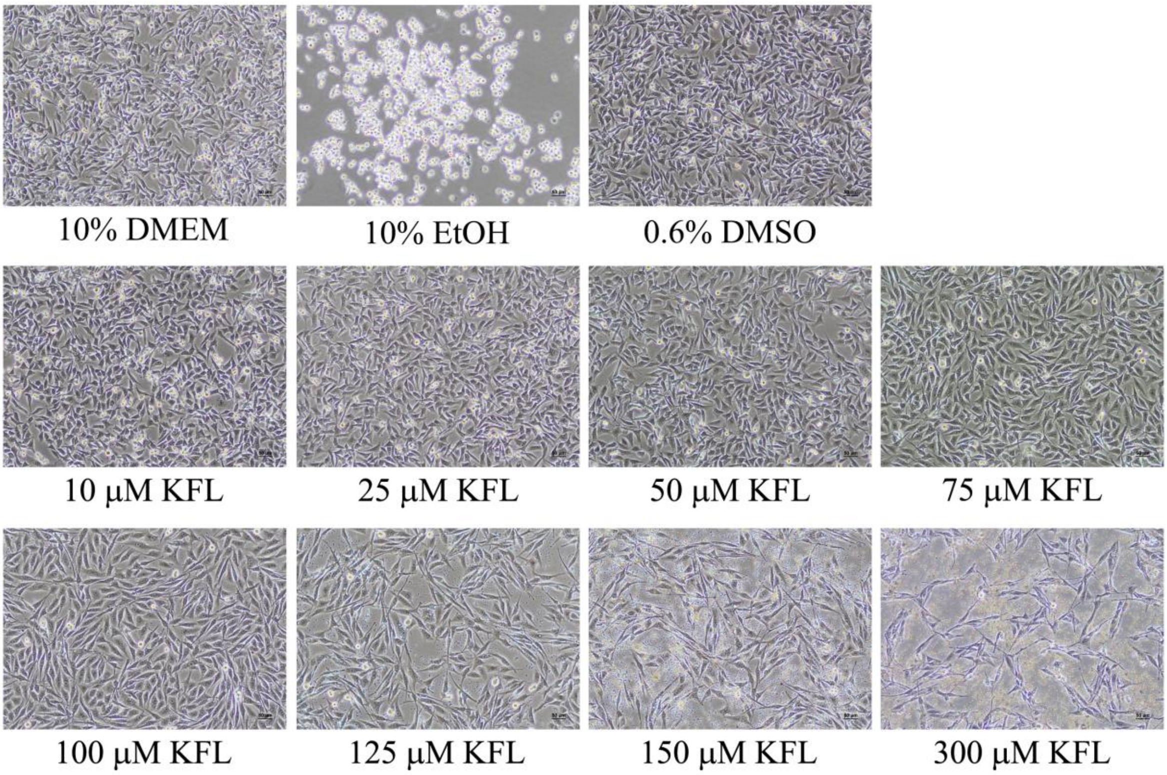

Supplemental Figure S3. Morphological changes of BHK-21 cells after treatment with kaempferol

BHK-21 cells were treated with various concentrations of DMSO or kaempferol for 24 hrs. The cytotoxicity of kaempferol on cell morphology was observed under the inverted microscope. Cells that were treated with 10% EtOH were used as a positive control. All experiments were undertaken independently in duplicate. Magnification: 200 X, Scale bar 20 µm. Enlargements of representative panels can be found in Supplementary Figures S27-37.

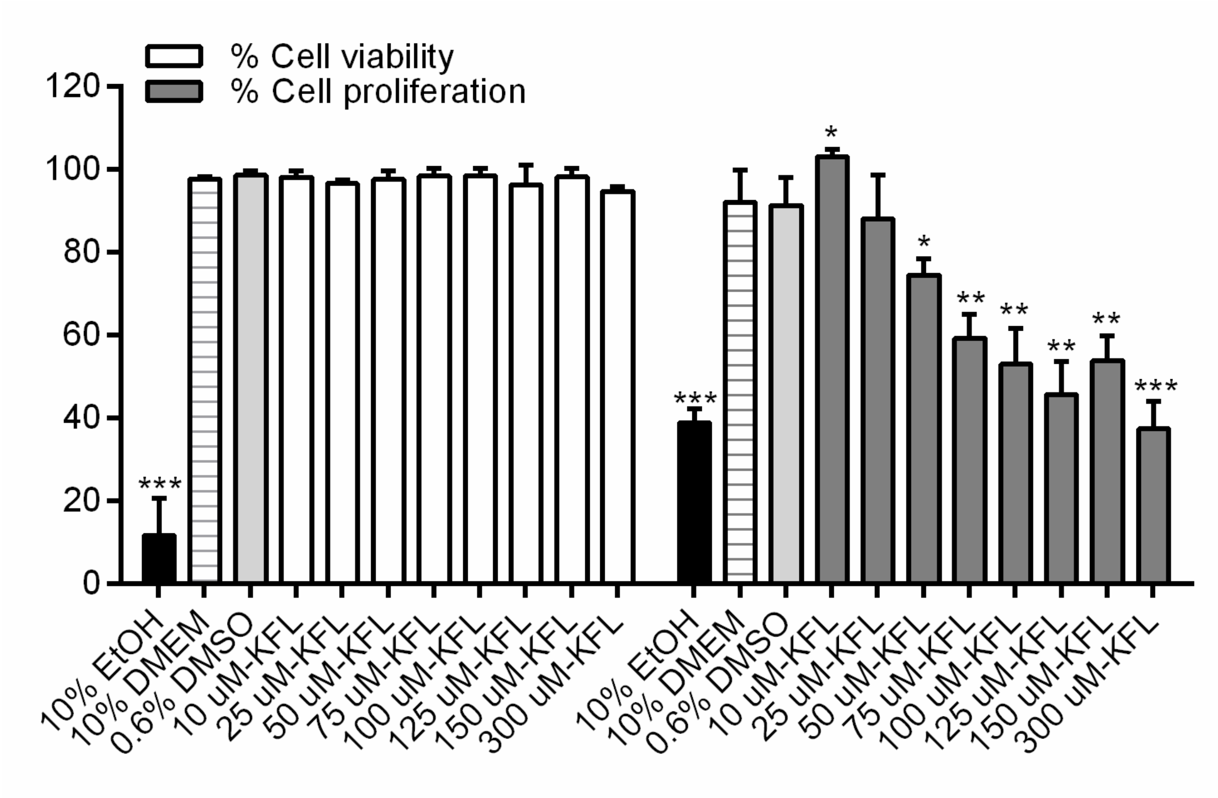

Supplemental Figure S4. Trypan blue staining and proliferation assay in BHK-21 cells.

BHK-21 cells were treated with various concentrations of DMSO or kaempferol for 24 hrs. The cytotoxicity of kaempferol was assessed by a trypan blue exclusion assay and by a cell proliferation assay (total cell count). Results are presented as percentage of cell viability from 4 replicates at 24 h post treatment. Negative (10% FBS in DMEM) and positive (10% EtOH) controls were included. Error bars represent mean ± SD *; p value < 0.05, **; p value <0.01 and ***; p value <0.001. All statistics were determined by comparison with the DMSO control.

Figure S5-26. Effect of Kaempferol on HEK293/17 cell morphology

S5. 10% DMEM

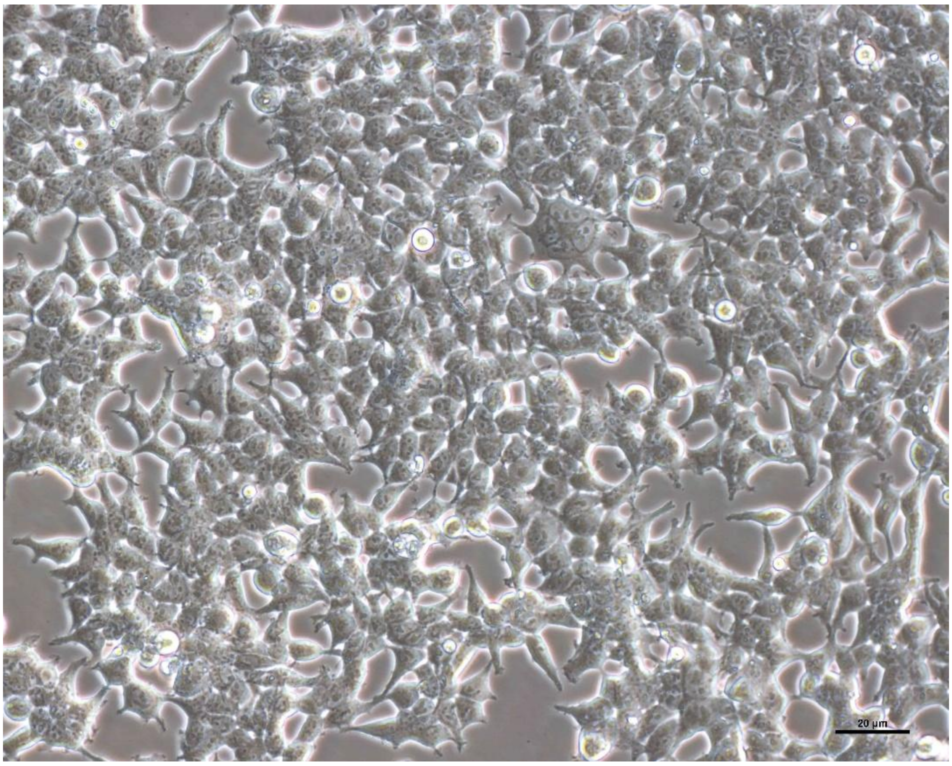

S6. 10% EtOH

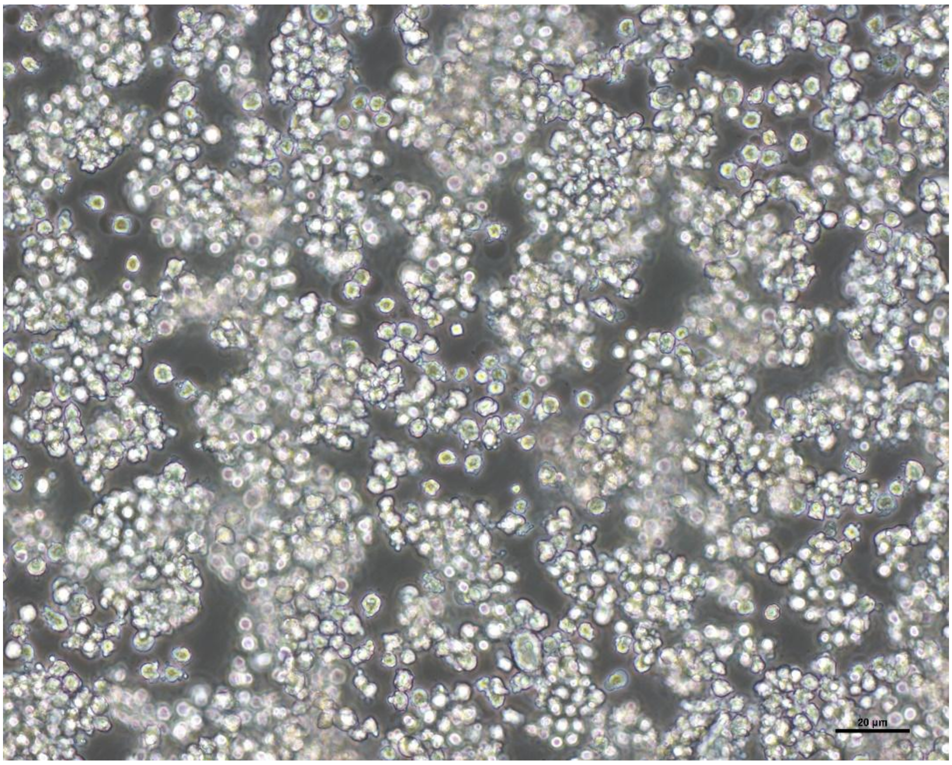

S7. 0.0002% DMSO

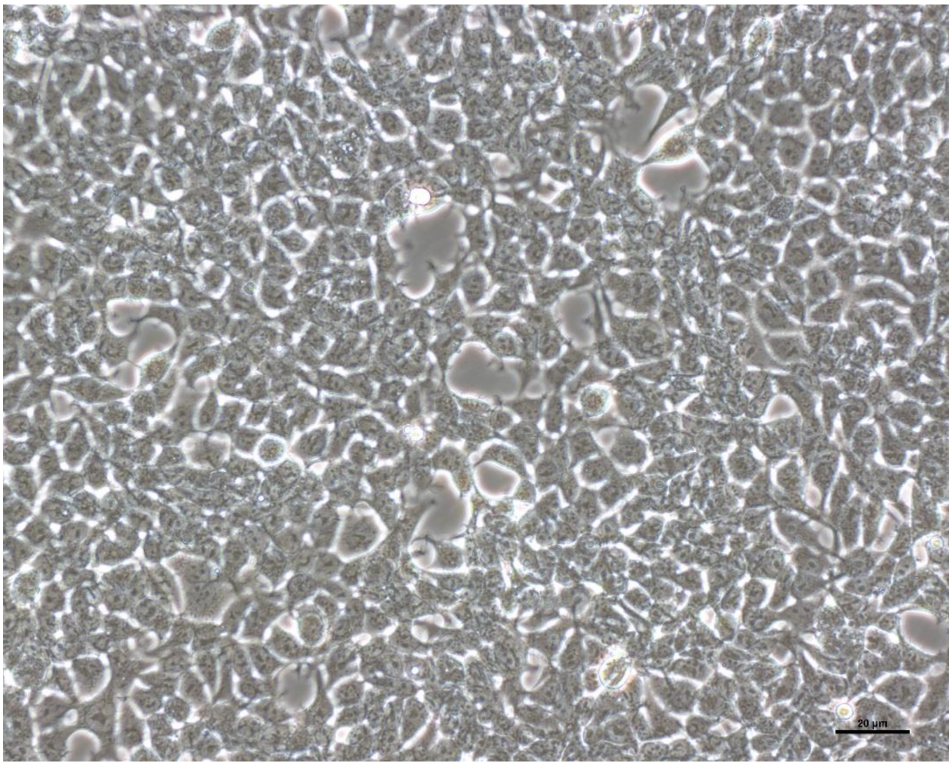

S8. 0.001% DMSO

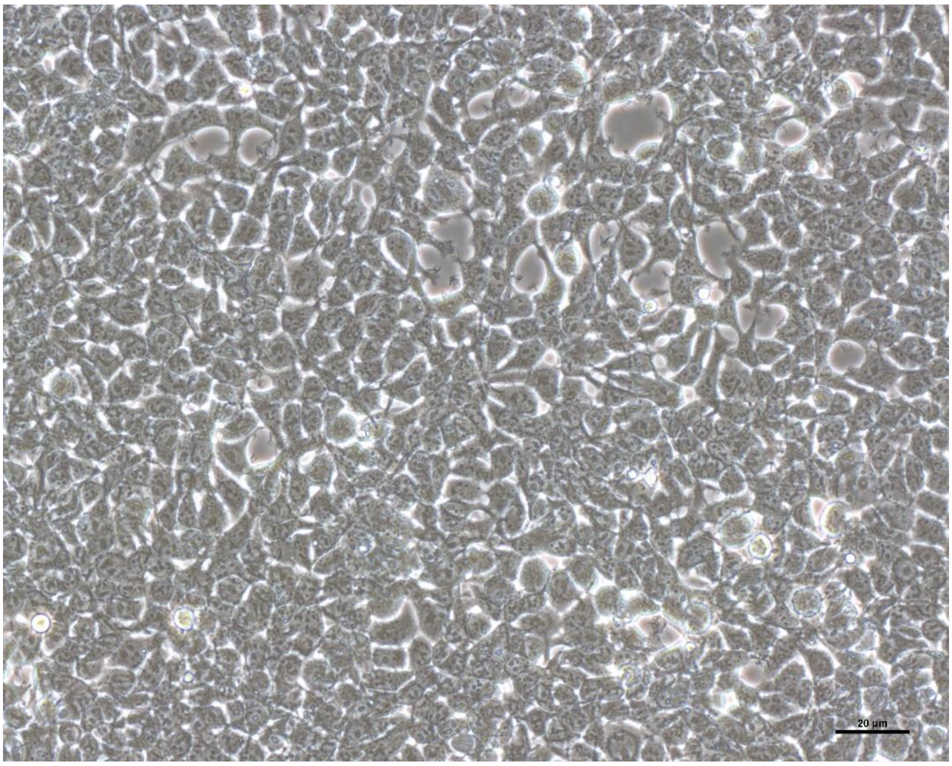

S9. 0.002% DMSO

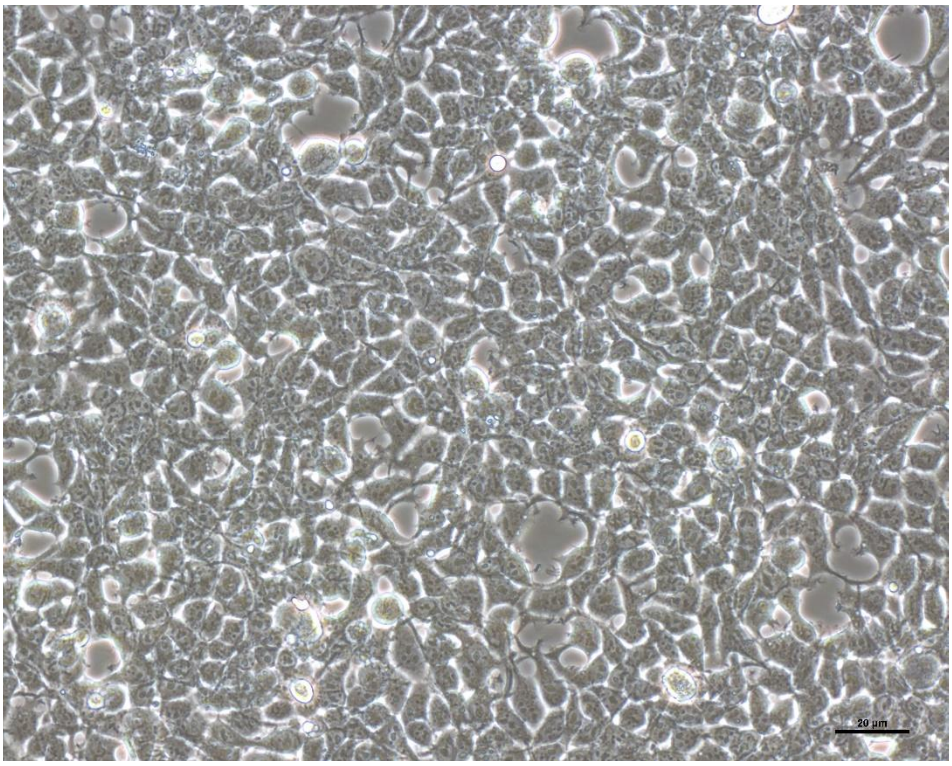

S10. 0.01% DMSO

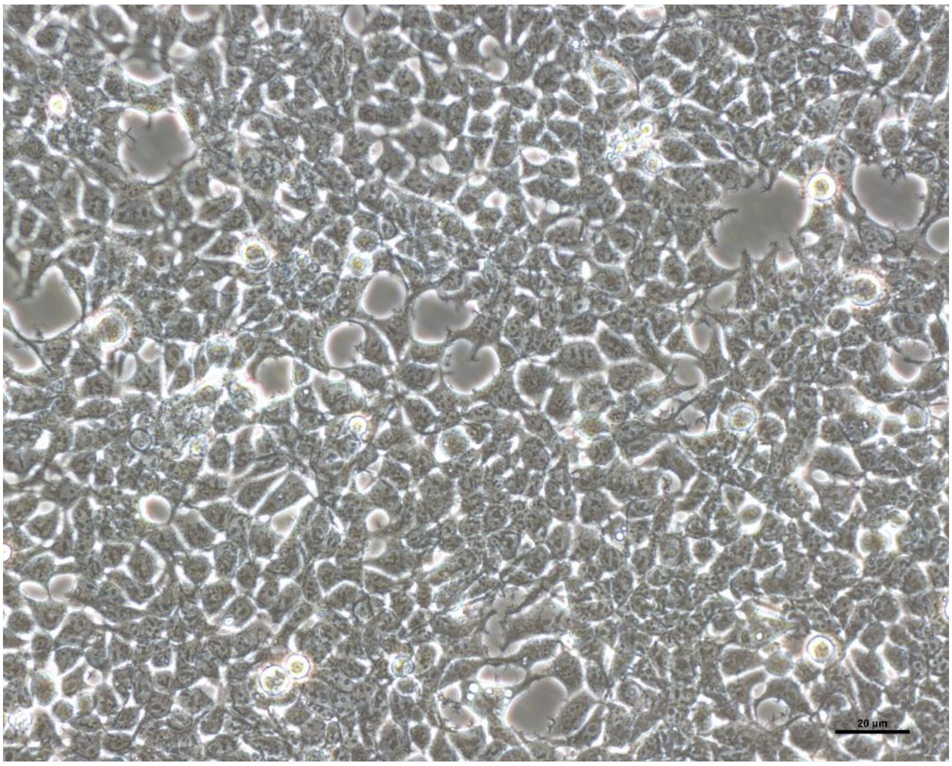

S11. 0.02% DMSO

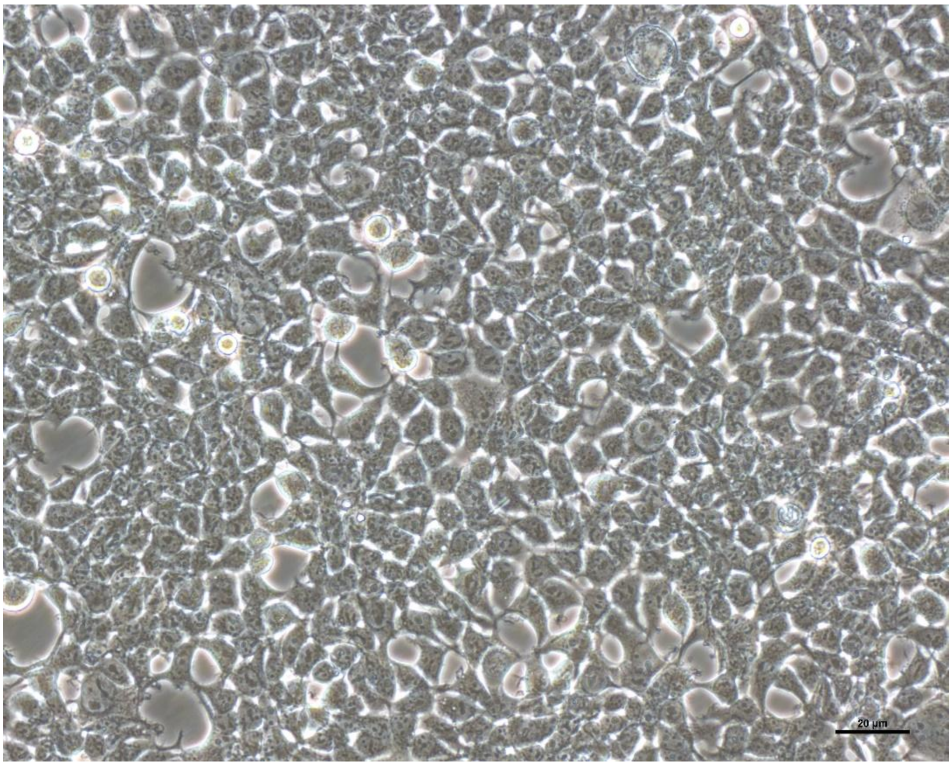

S12. 0.05% DMSO

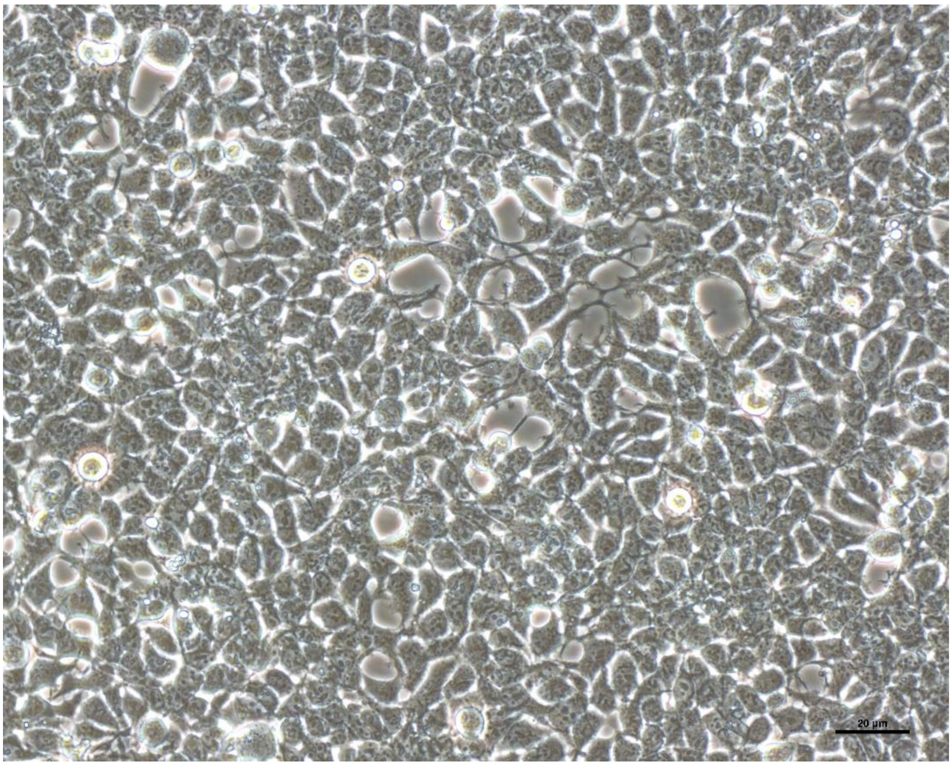

S13. 0.1% DMSO

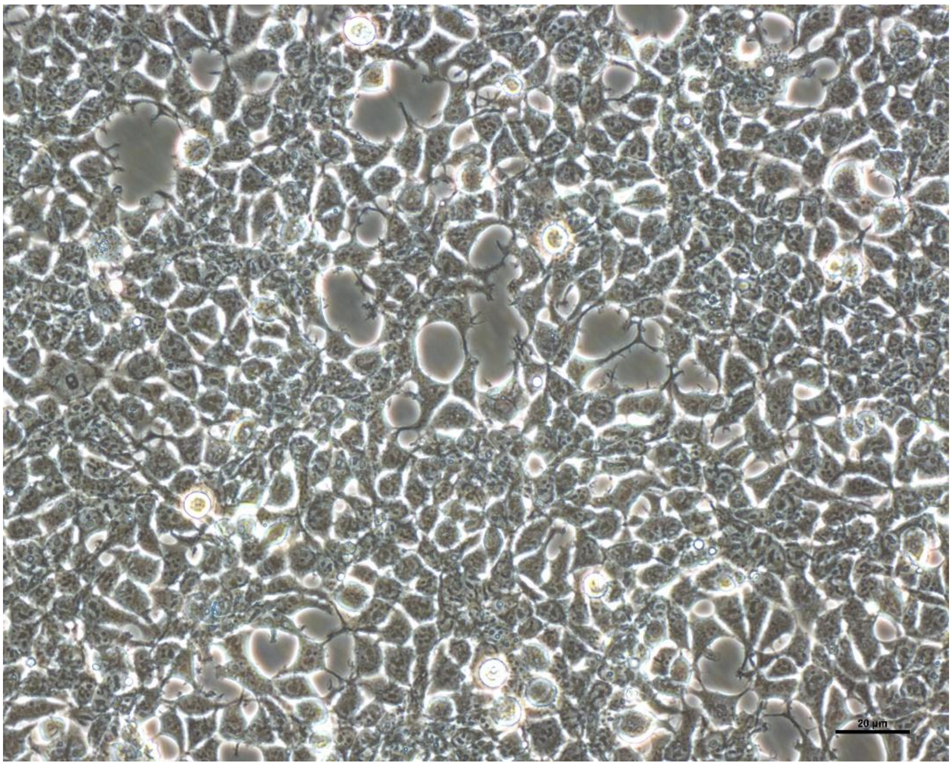

S14. 0.2% DMSO

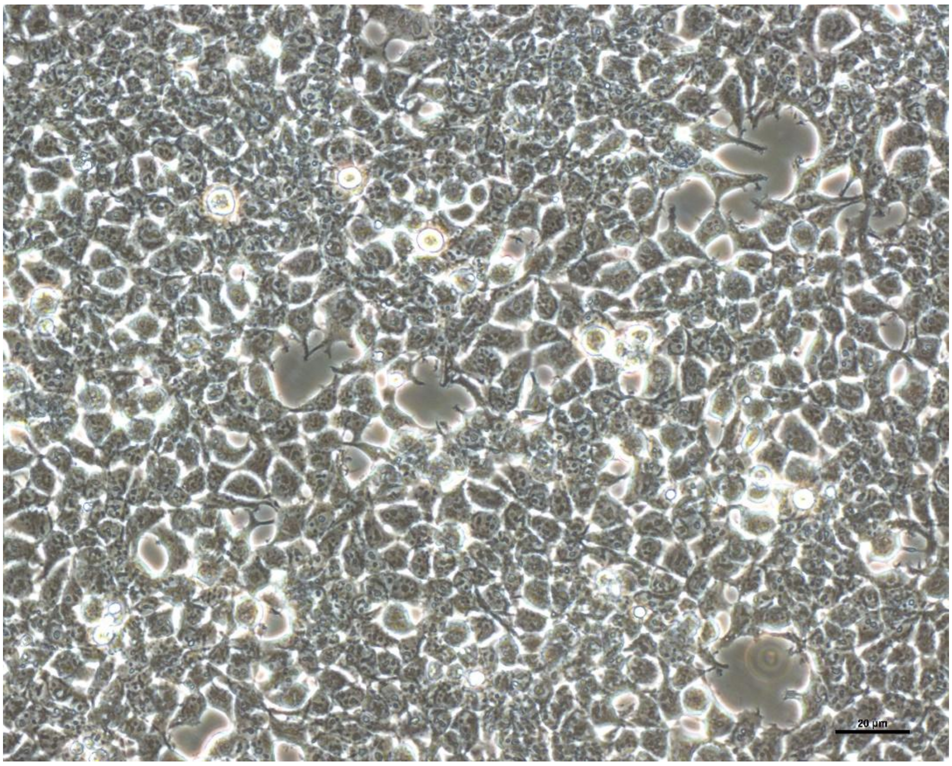

S15. 0.4% DMSO

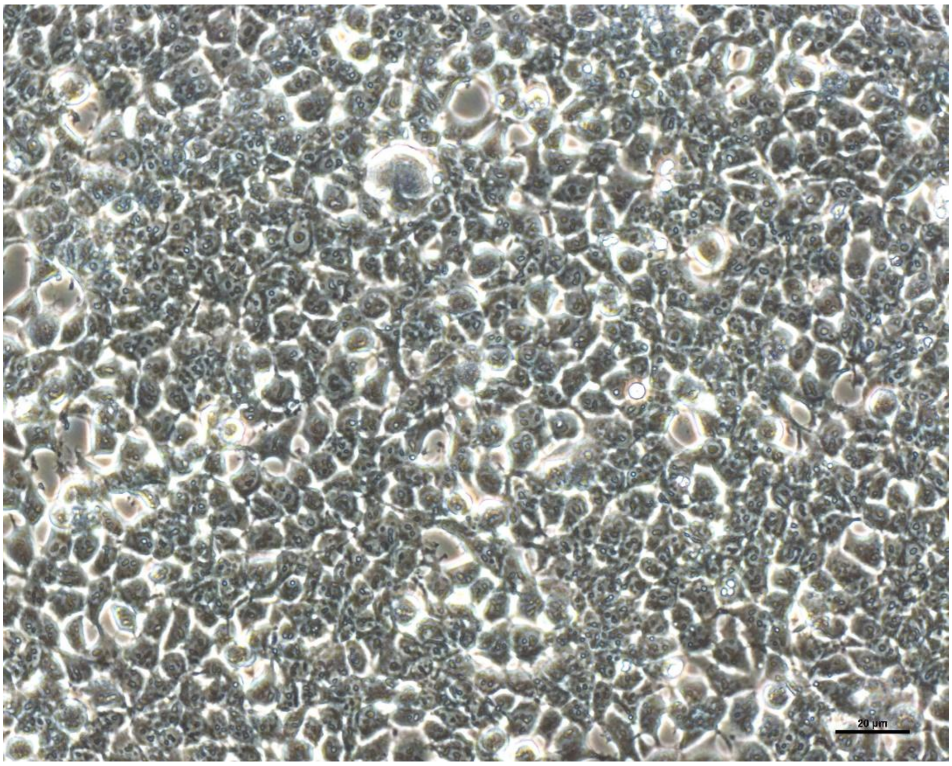

S16. 0.8% DMSO

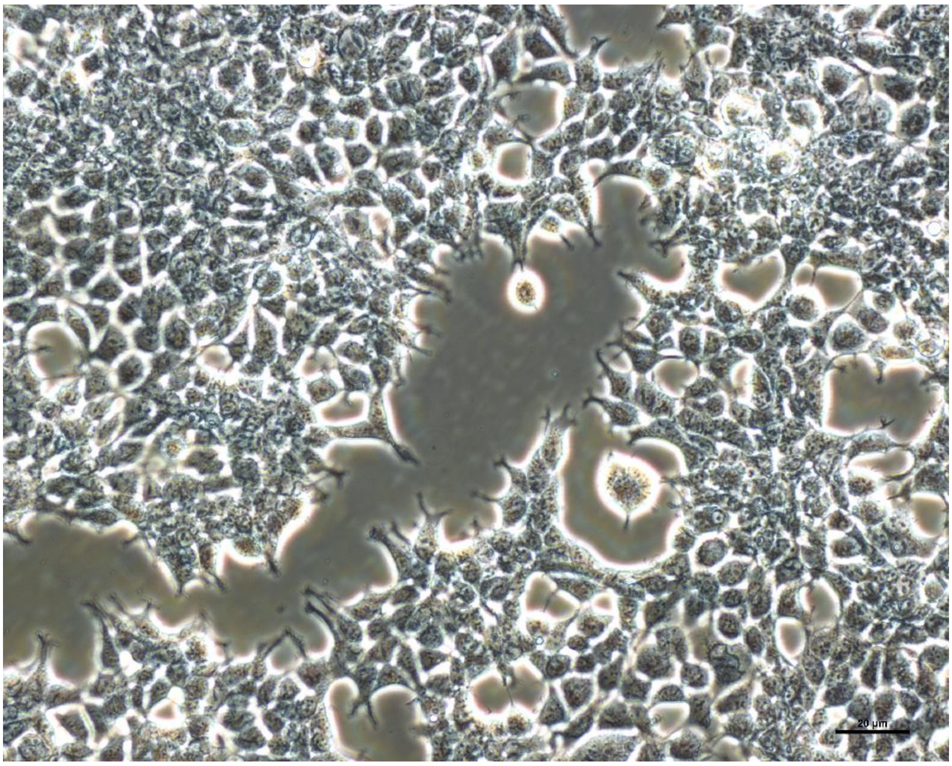

S17. 0.1 µM Kaempferol

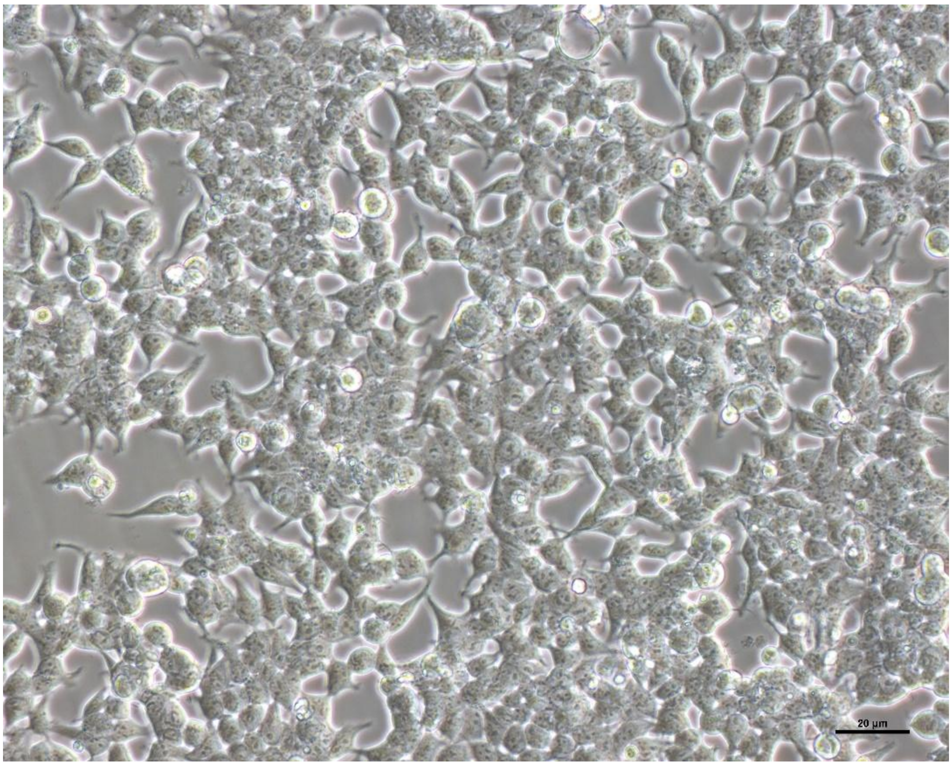

S18. 0.5 µM Kaempferol

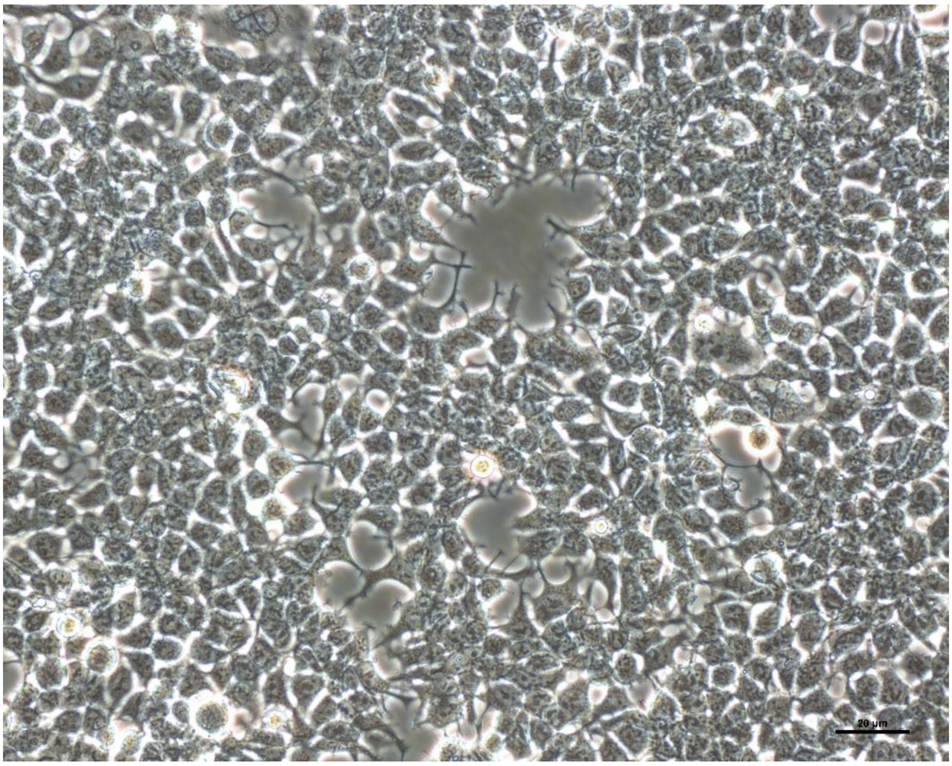

S19. 1 µM Kaempferol

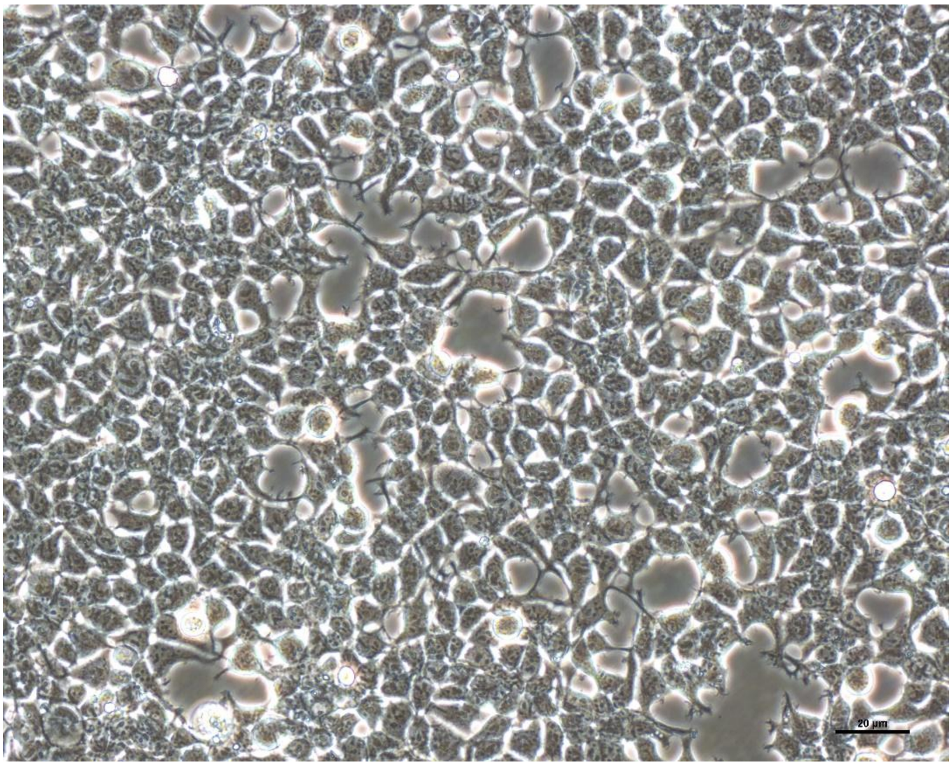

S20. 5 µM Kaempferol

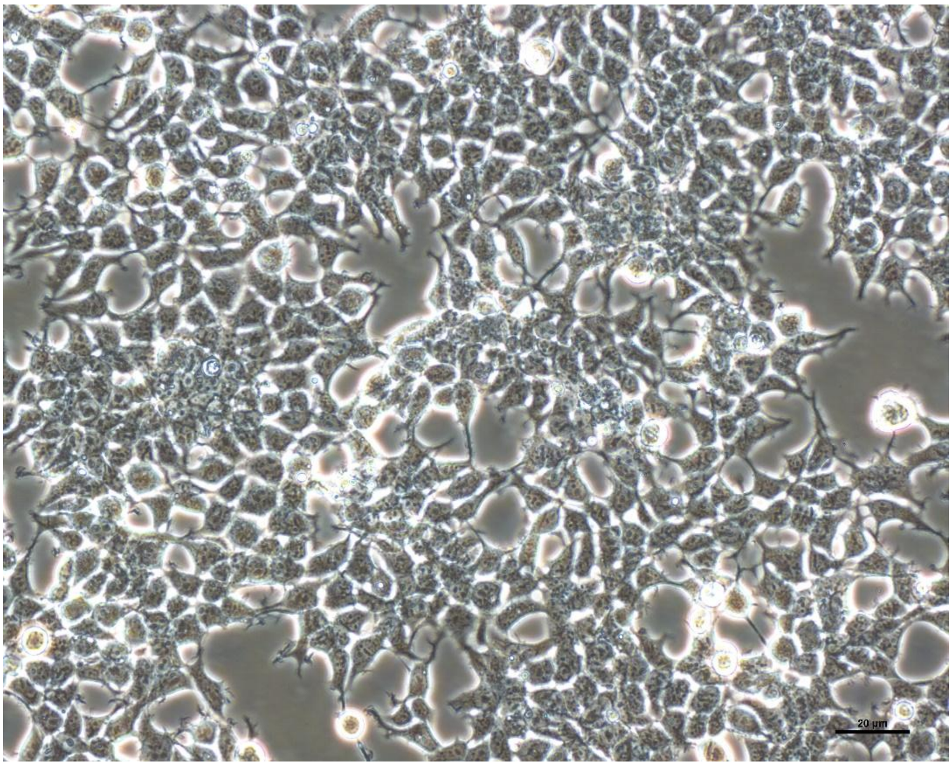

S21. 10 µM Kaempferol

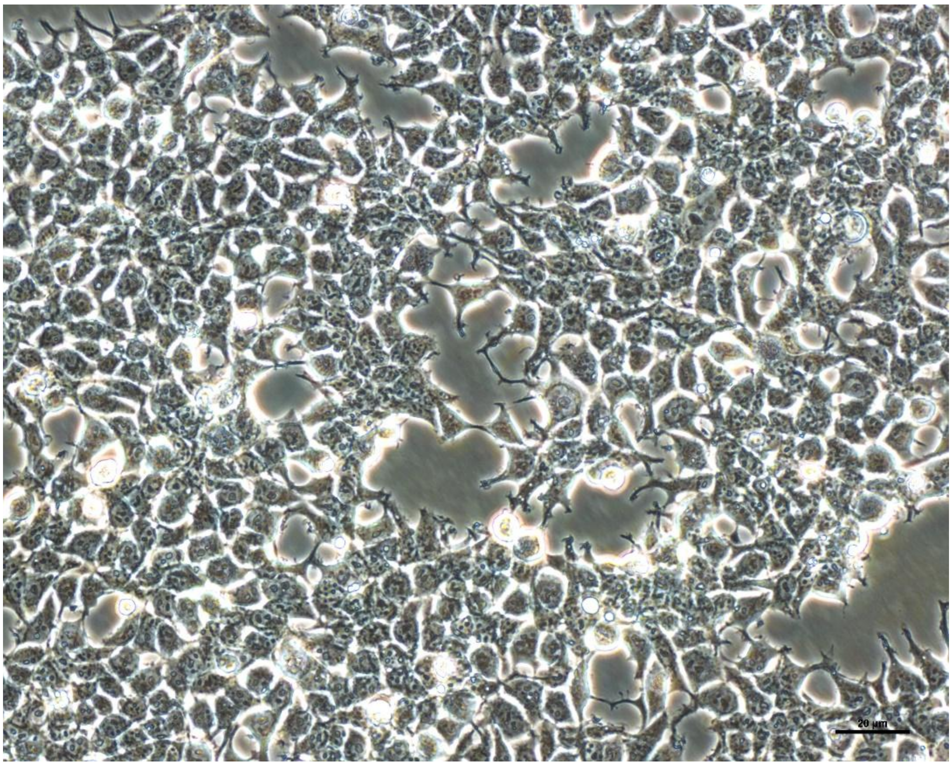

S22. 25 µM Kaempferol

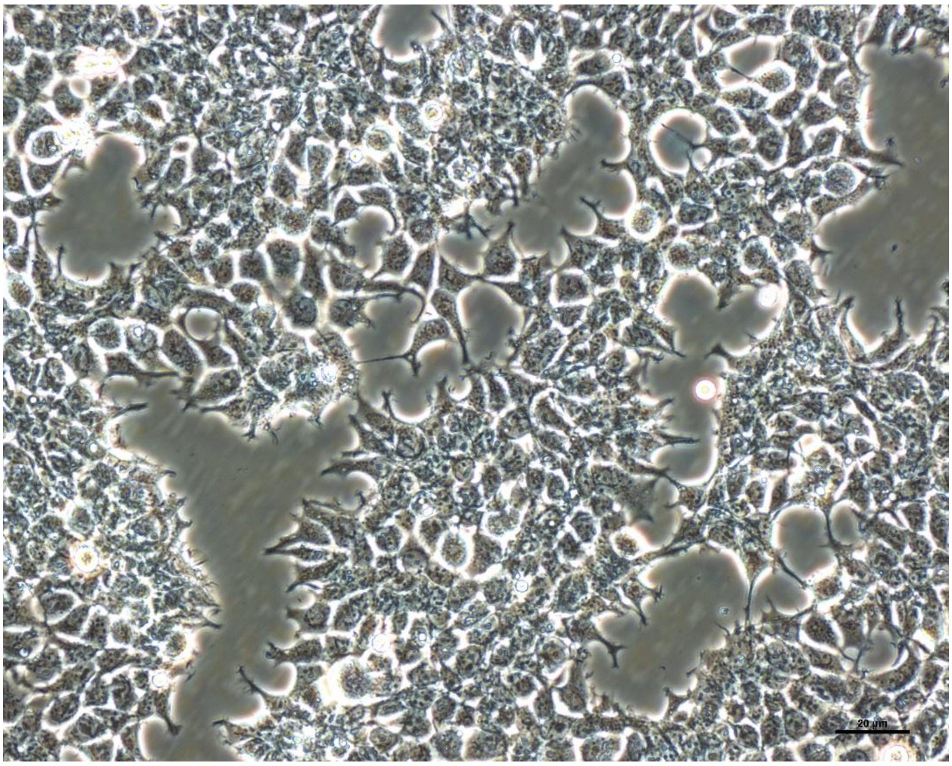

S23. 50 µM Kaempferol

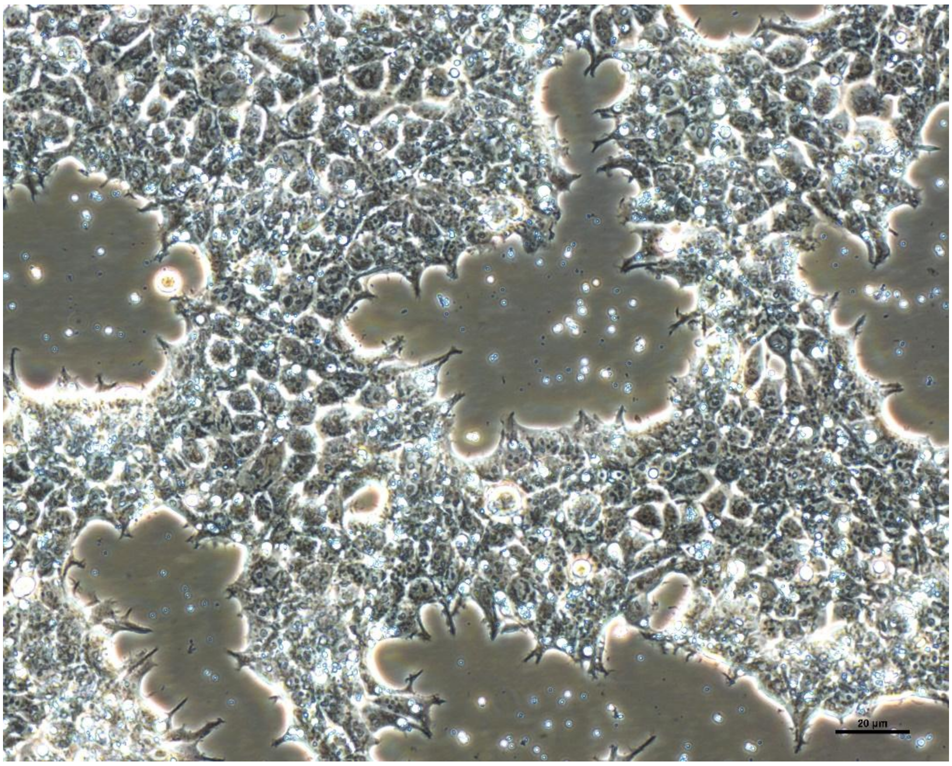

S24. 100 µM Kaempferol

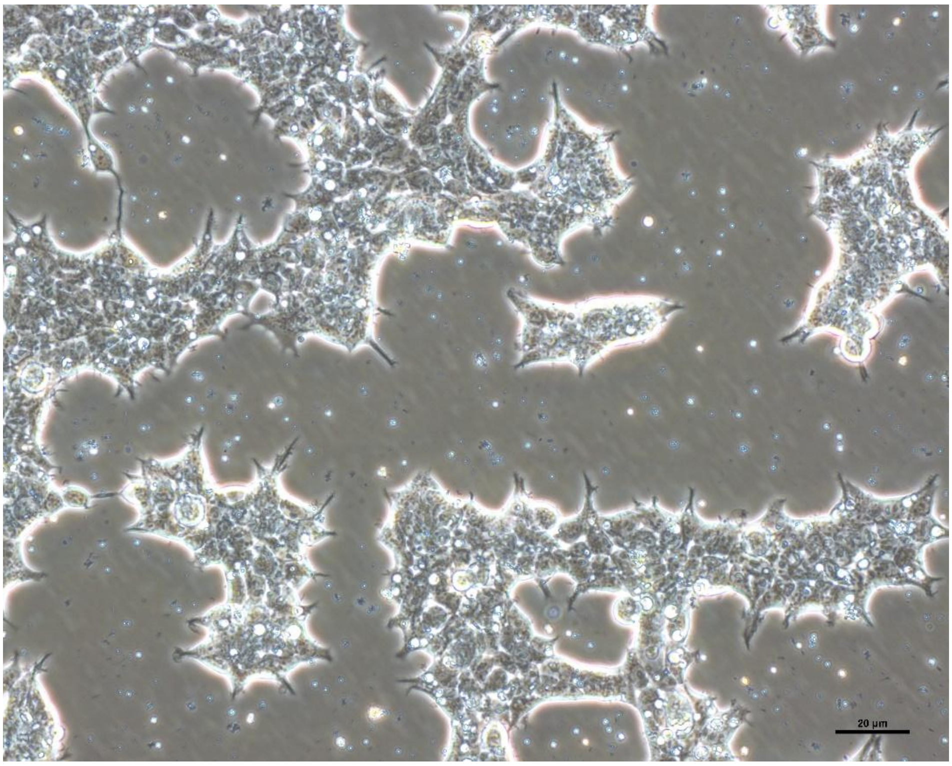

S25. 200 µM Kaempferol

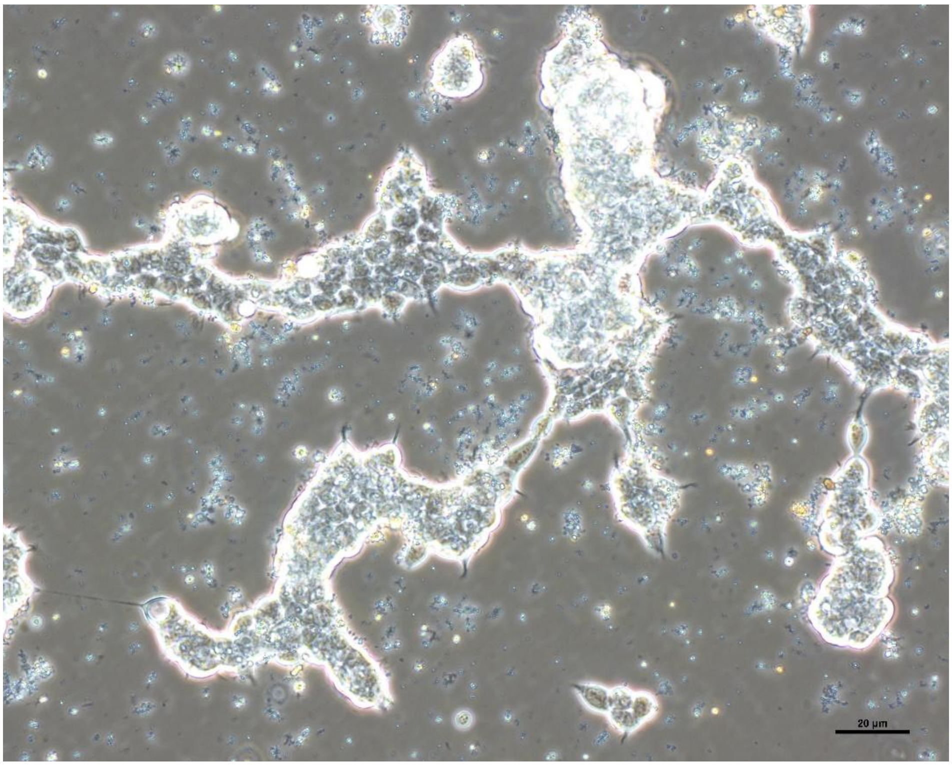

S26. 400 µM Kaempferol

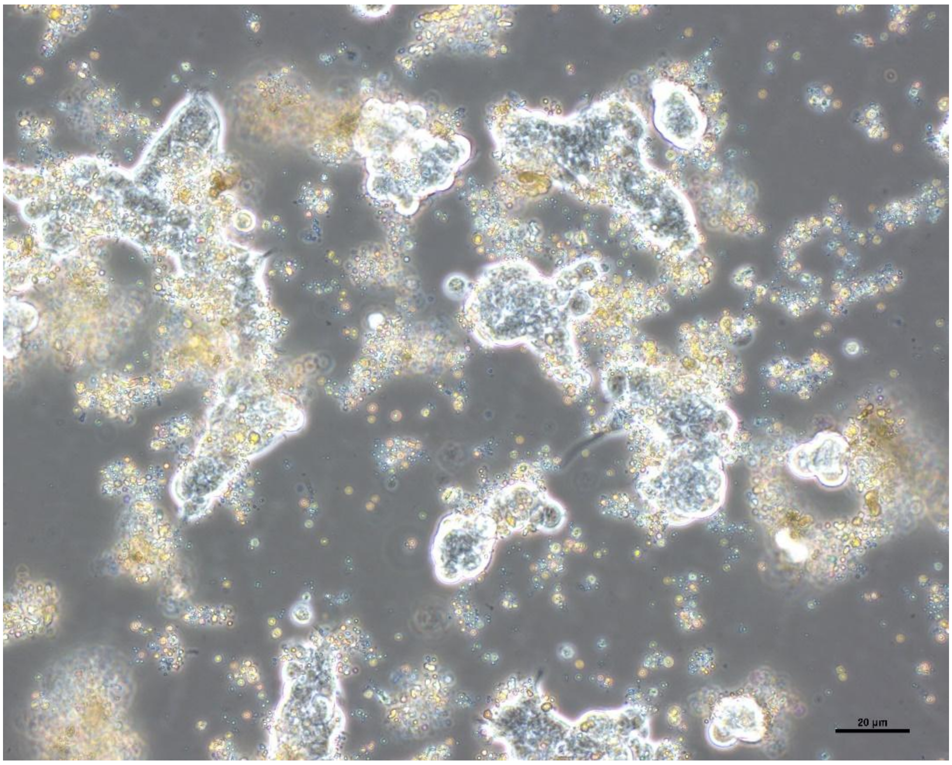

Figure S27-37. Effect of Kaempferol on BHK-21 cell morphology

S27. 10% DMEM

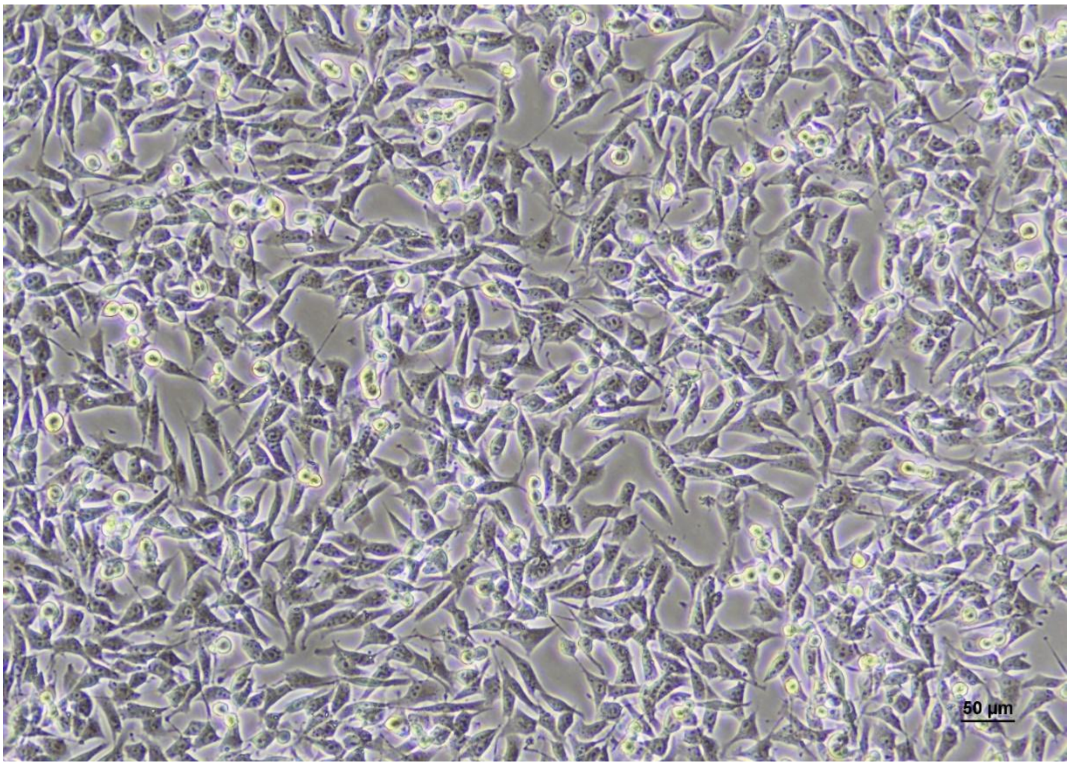

S28. 10% EtOH

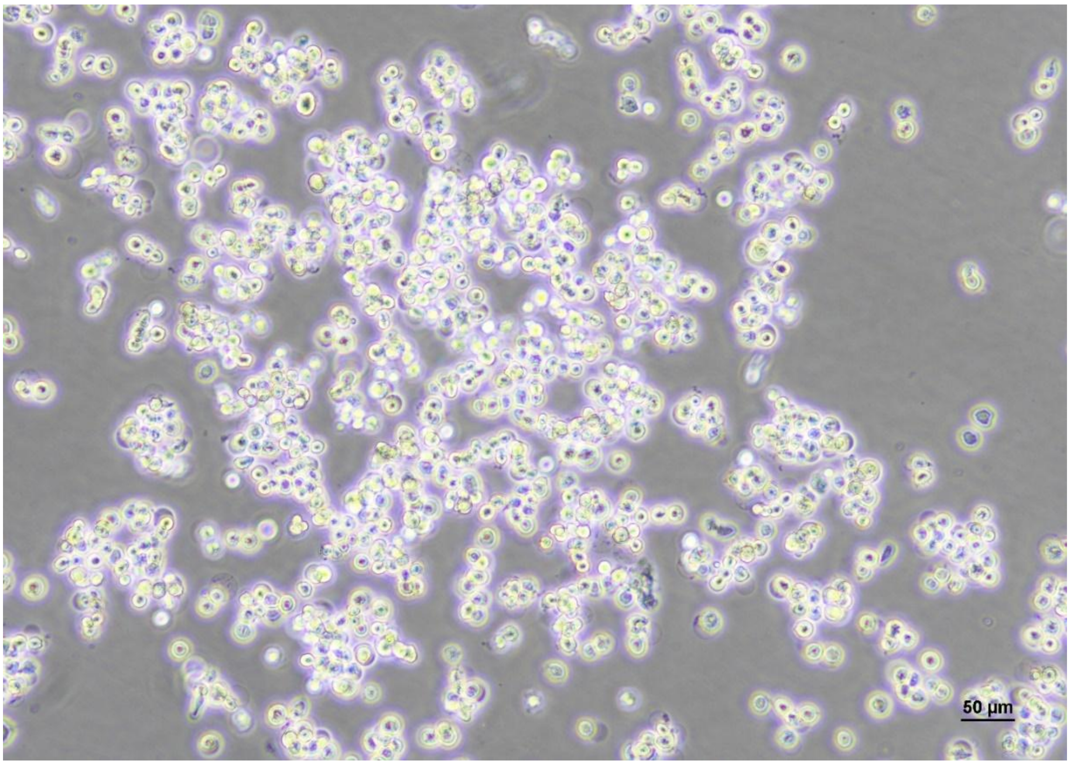

S29. 0.6% DMSO

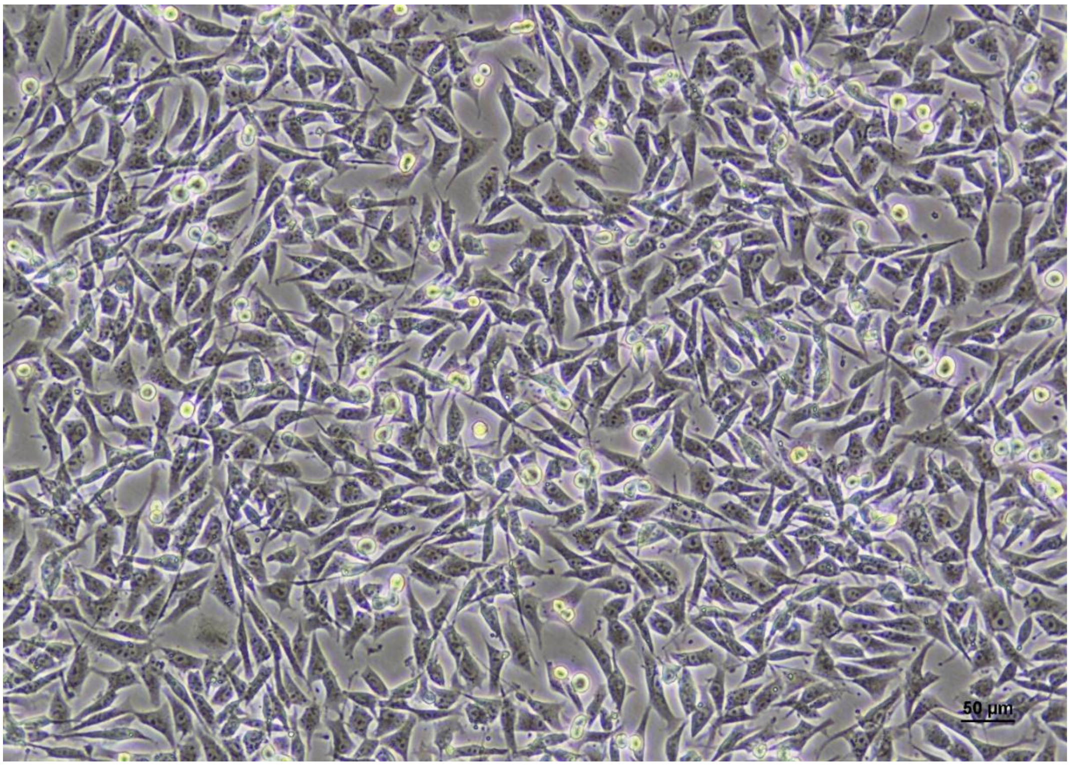

S30. 10 µM Kaempferol

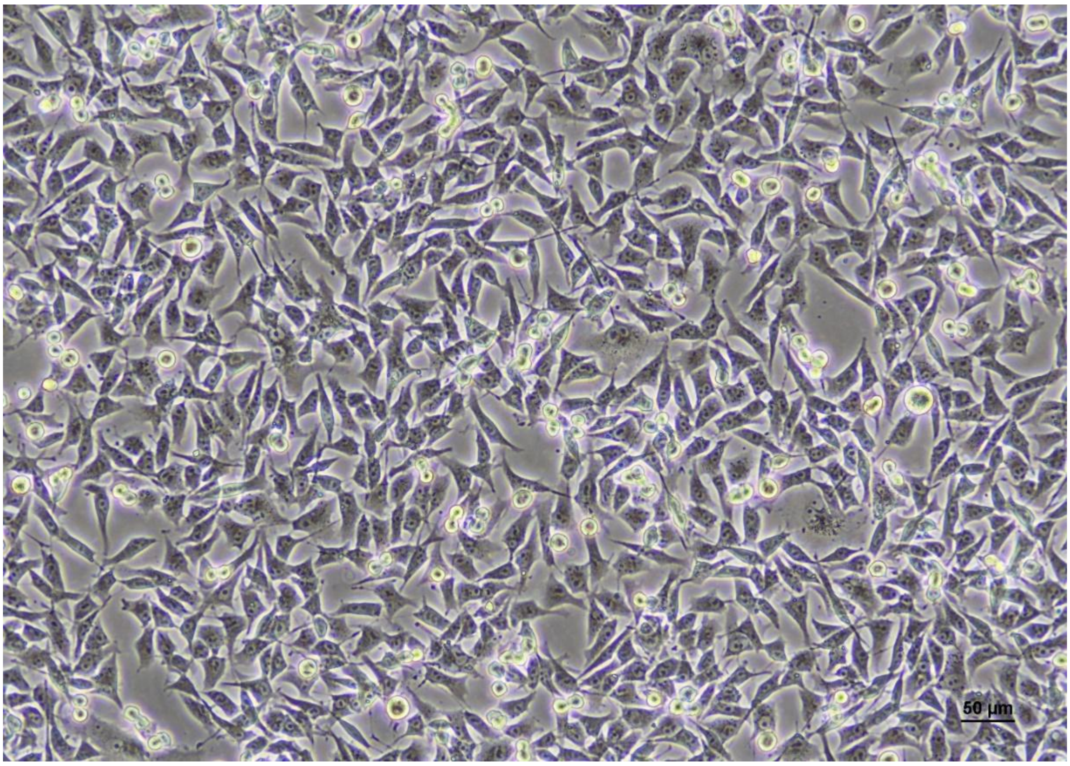

S31. 25 µM Kaempferol

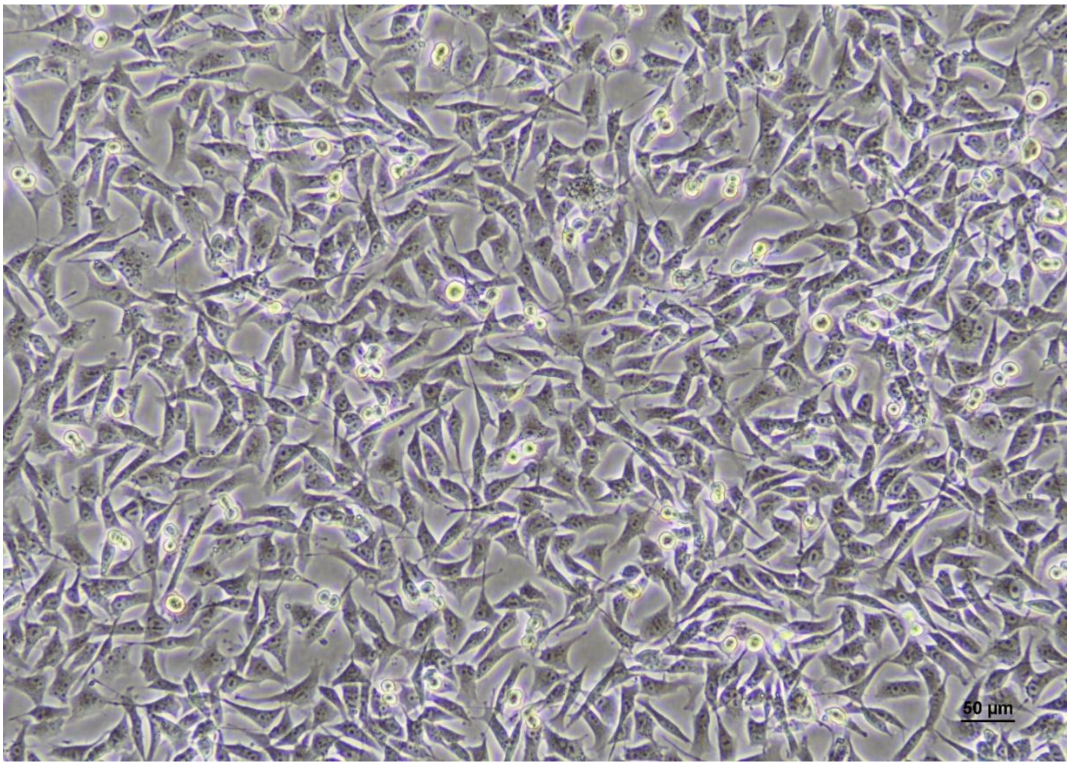

S32. 50 µM Kaempferol

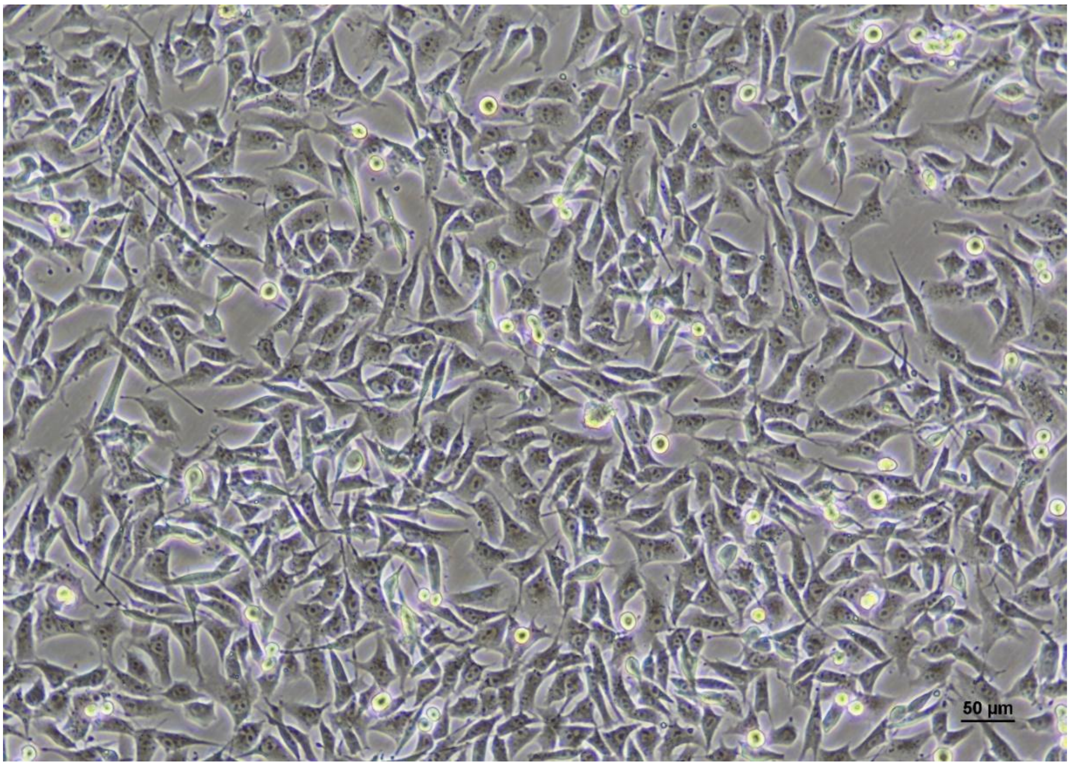

S33. 75 µM Kaempferol

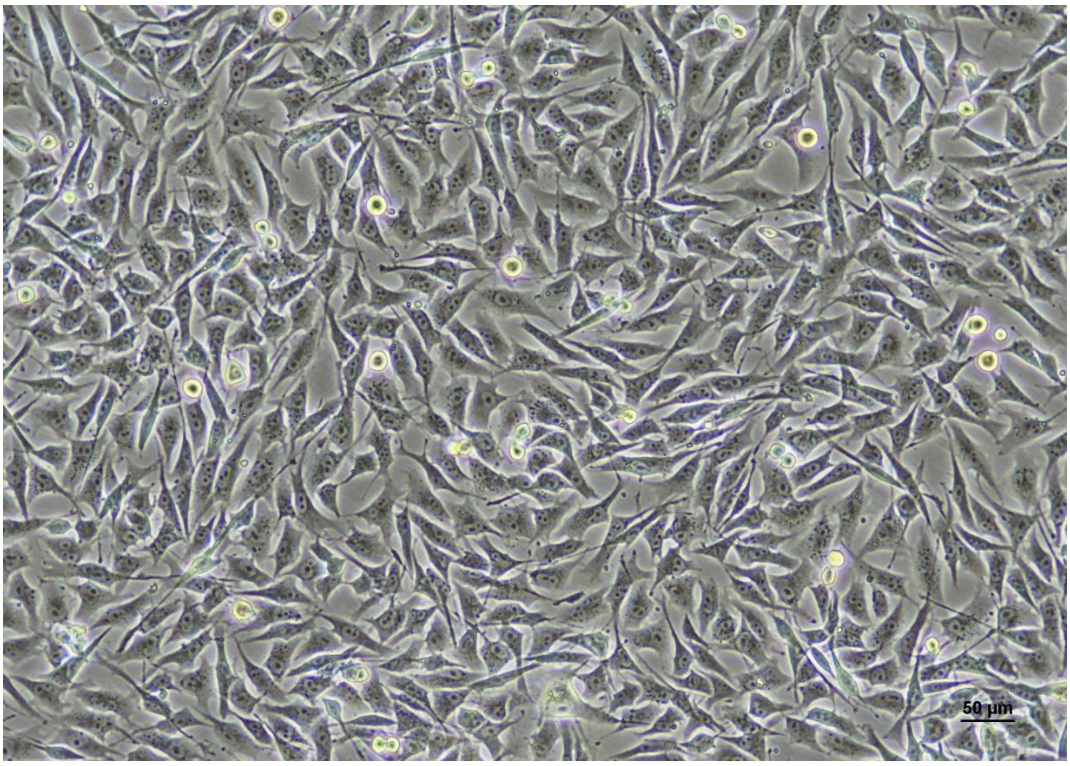

S34. 100 µM Kaempferol

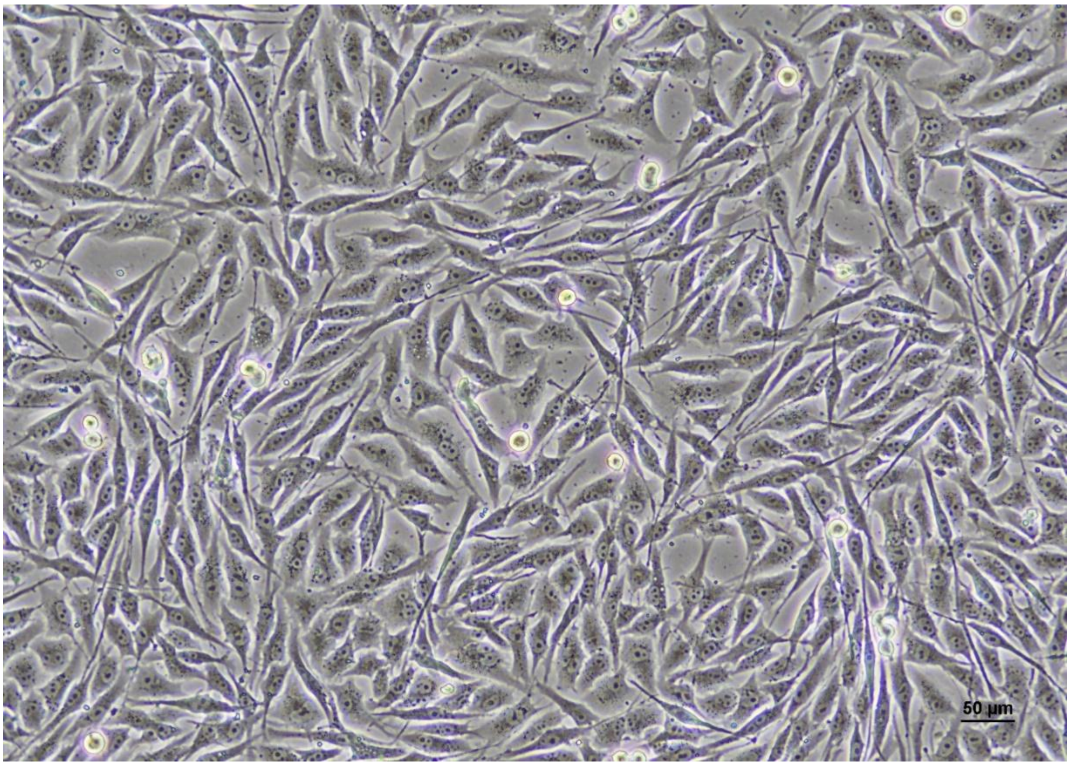

S35. 125 μM Kaempferol

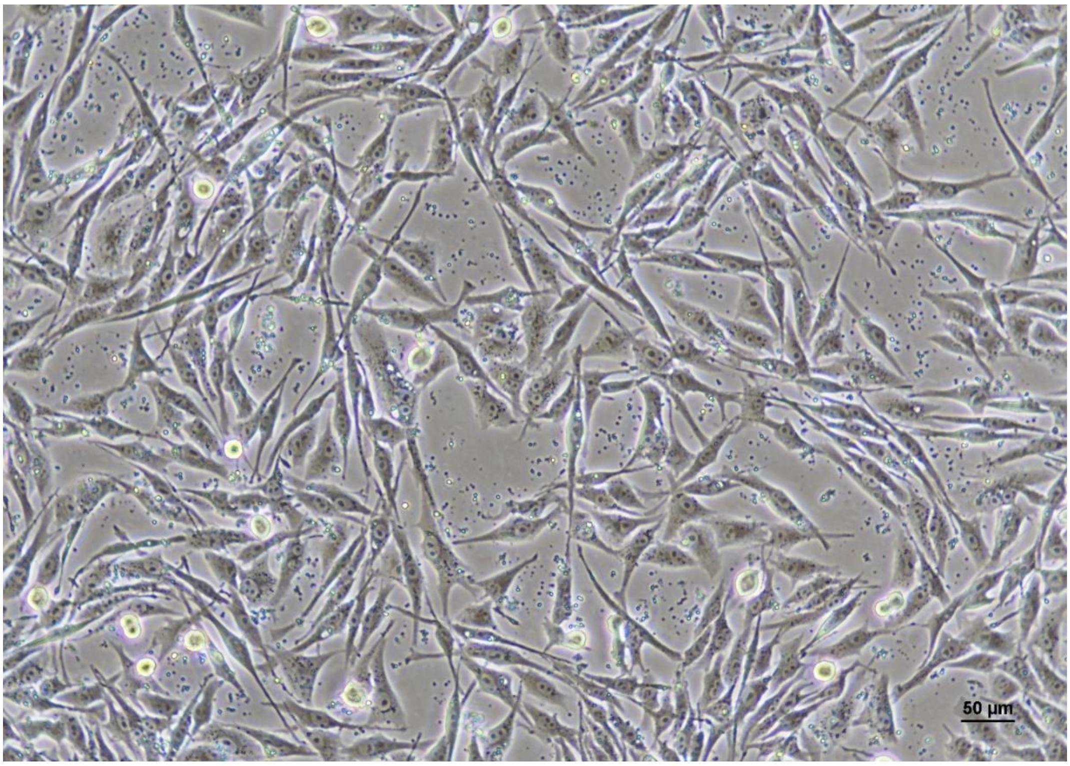

S36. 150 μM Kaempferol

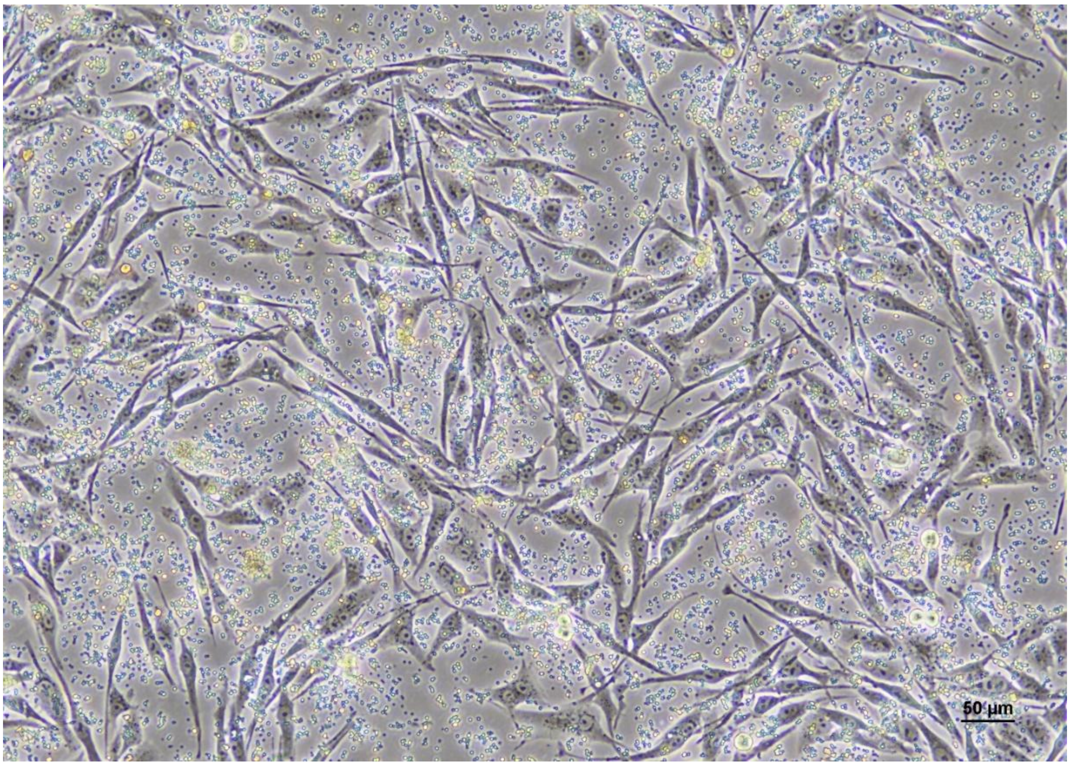

S37. 300 µM Kaempferol

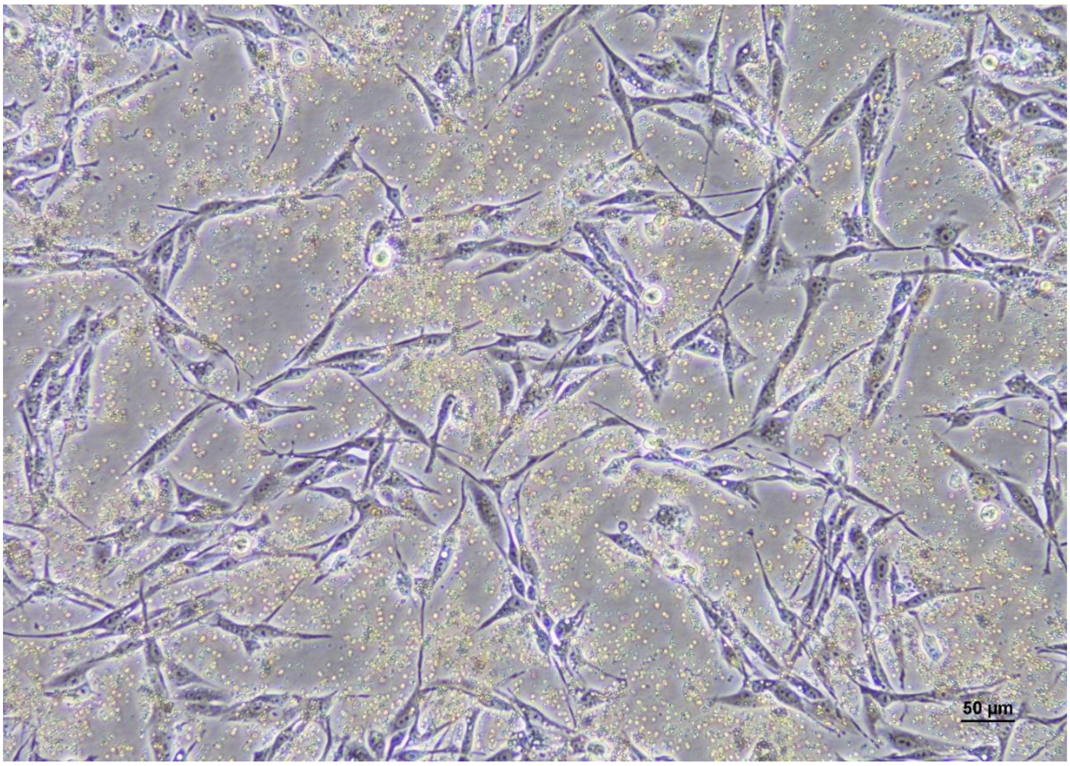

Supplementary Table 1. Primer sequences.

Primer	Sequence (5'-3')
GRP78-F	AGACTTTGACCAGCGTGTCATGGA
GRP78-R	TTCAAATTTGGCCCGAGTCAGGGT
GAPDH-F	GAACATCATCCCTGCCTCTAC
GAPDH-R	CCTGCTTCACCACCTTCTT
β actin-F	GAAGATGACCCAGATCATGT
β actin-R	ATCTCTTGCTCGAAGTCCAG

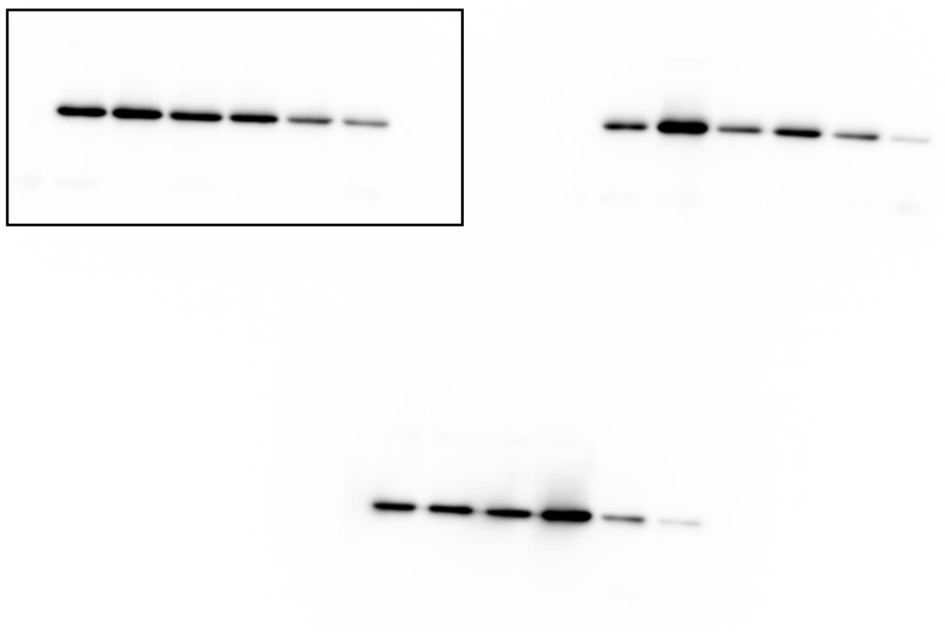

E protein of JEV with treatment of kamepferol

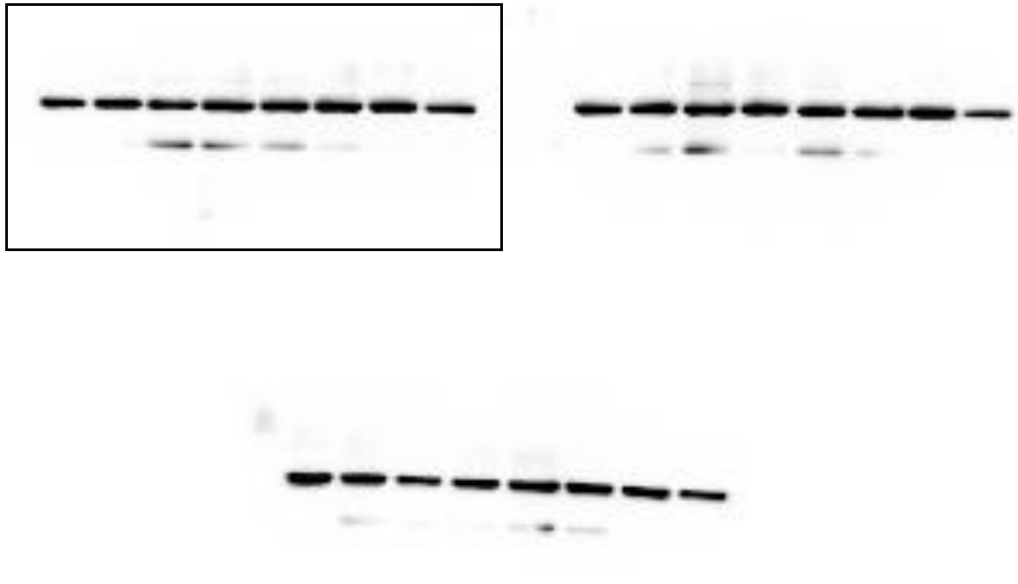

HSP70 of BHK-21 cells infected by JEV followed by treatment with kaempferol

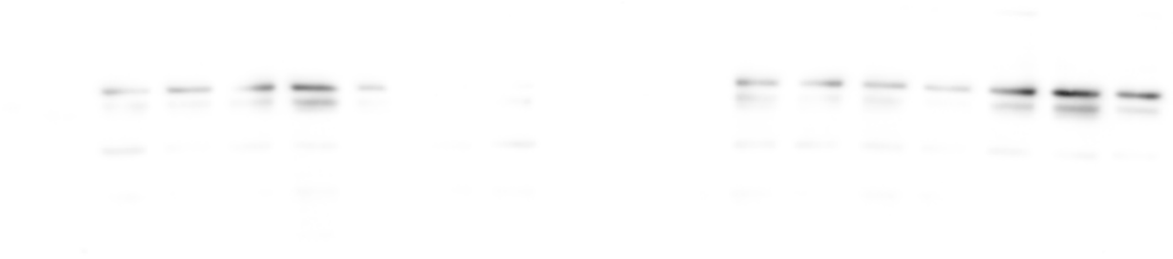

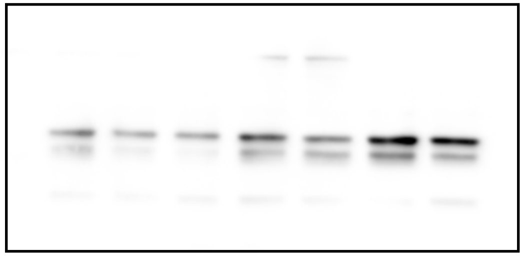

E protein of DENV with treatment of kamepferol

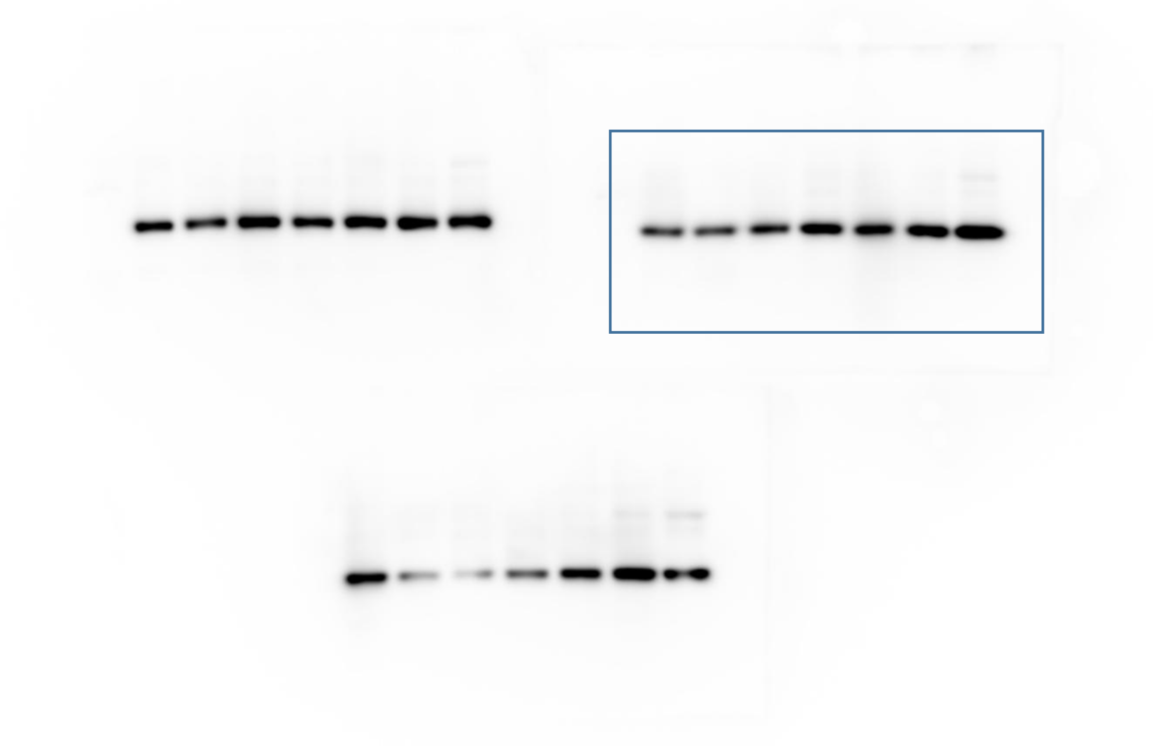

NS1 protein of DENV 2 treated with kaempferol

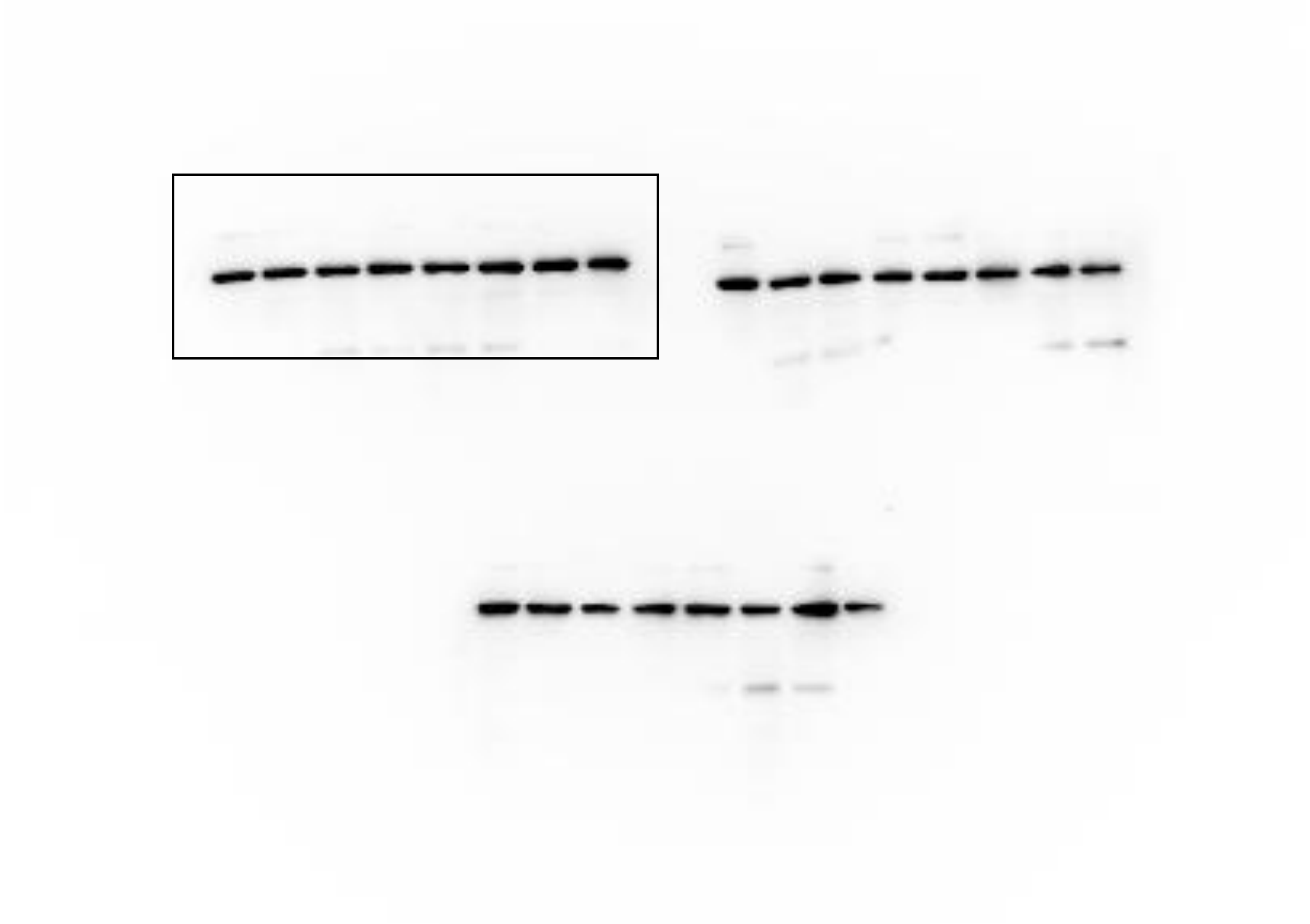

HSP70 of BHK-21 cells infected by DENV followed by treatment with kaempferol

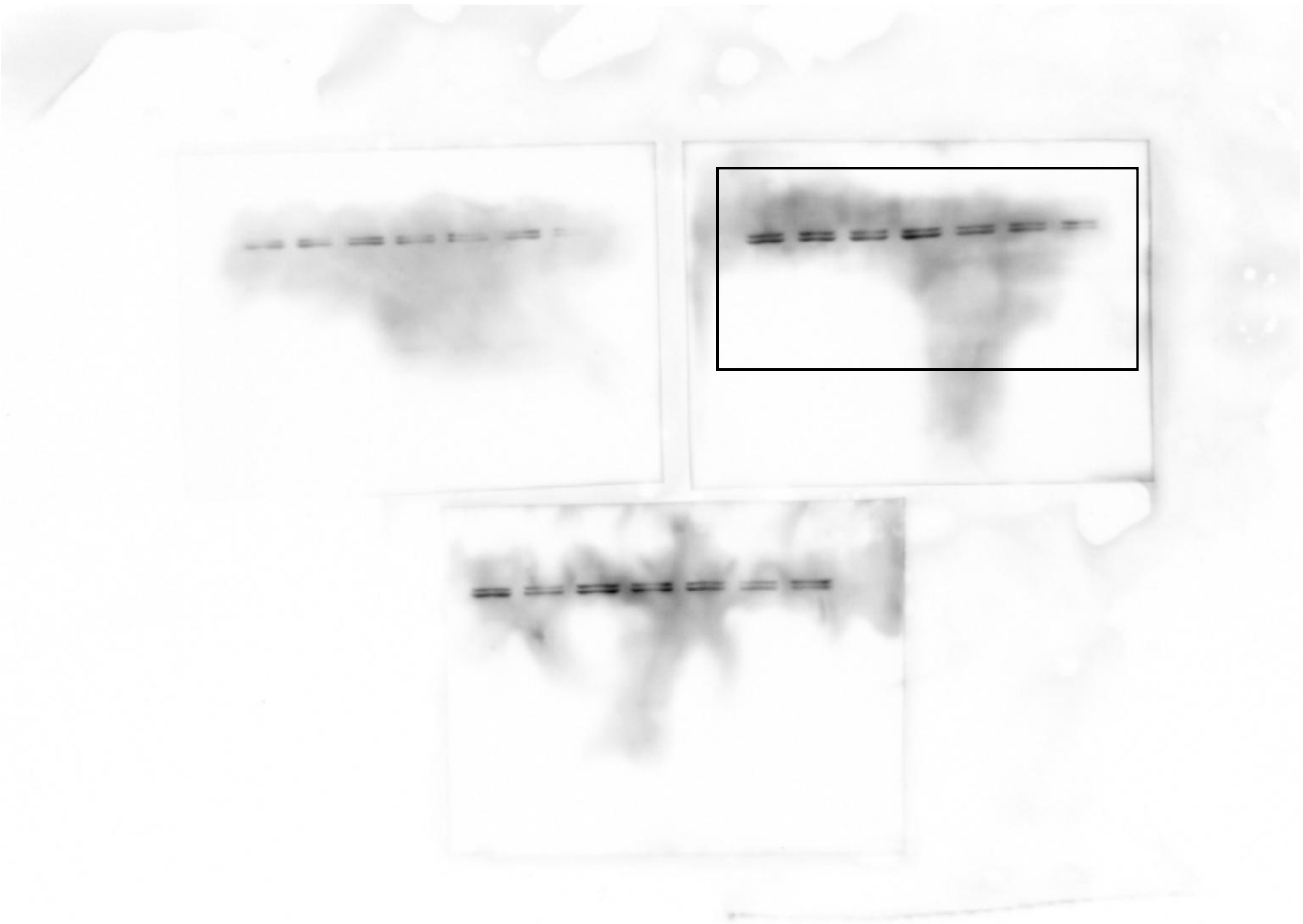

GRP 78 for control

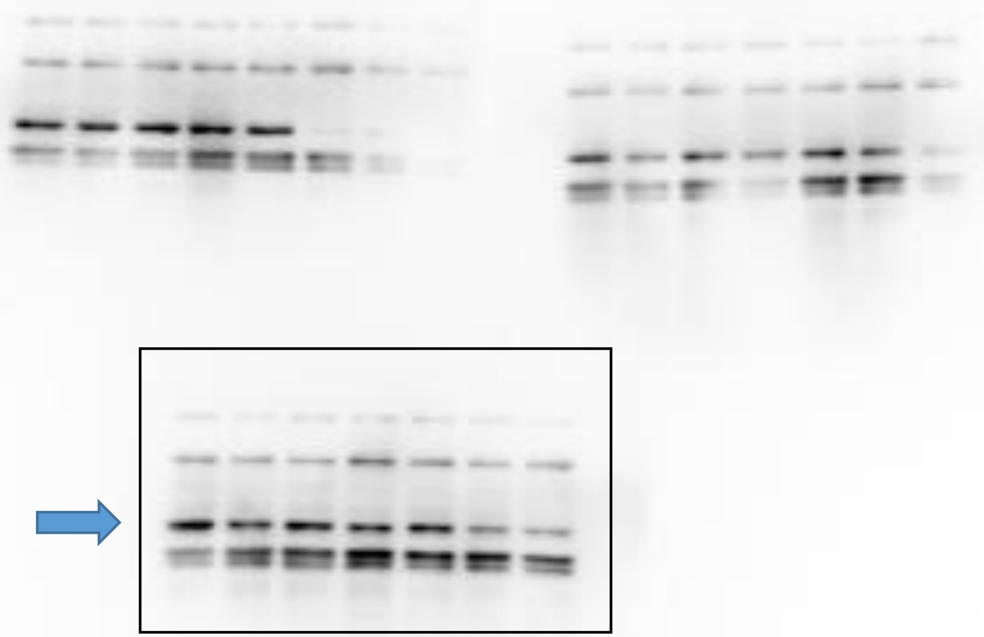

Actin for control

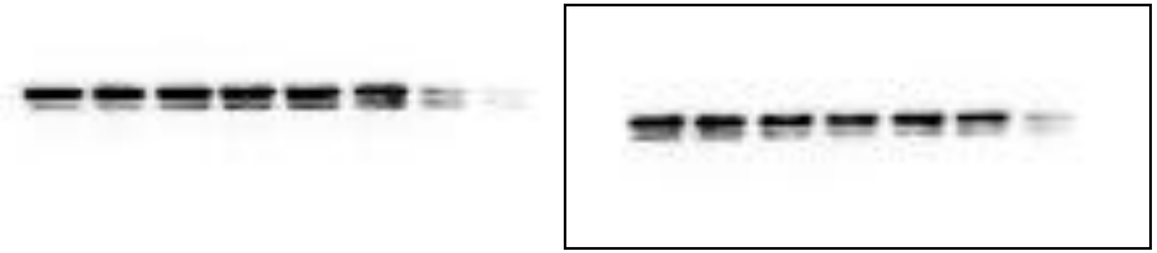

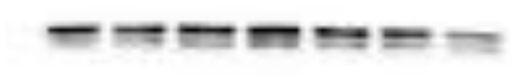

GAPDH for control

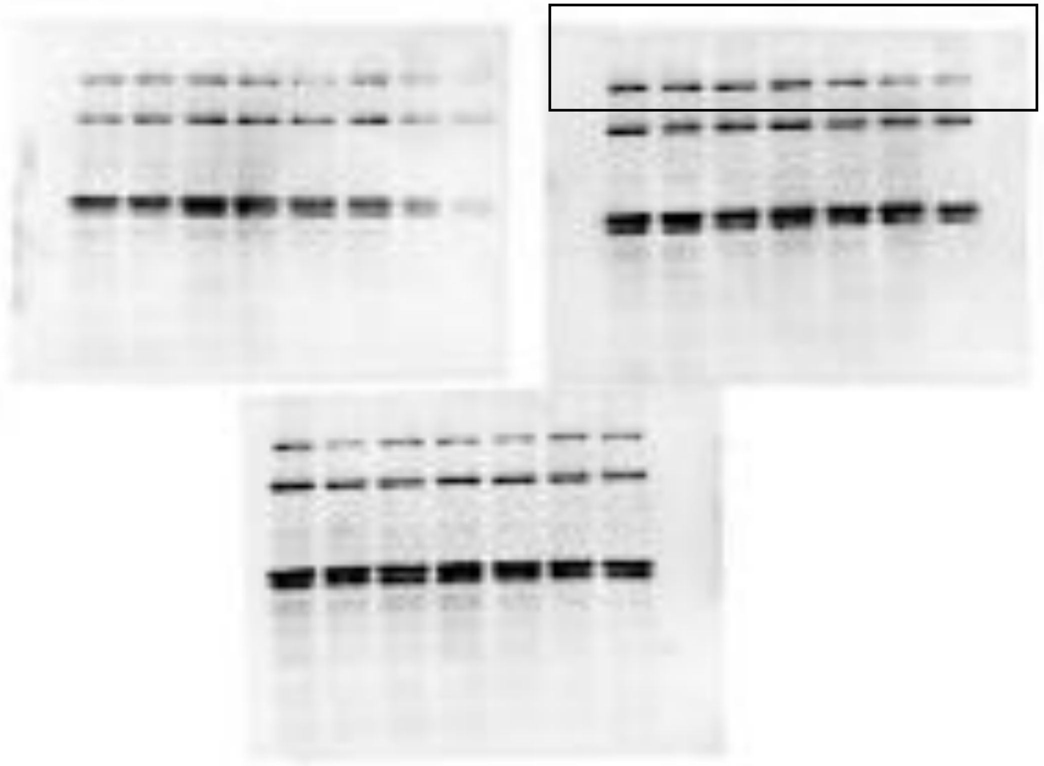

Vinculin for control

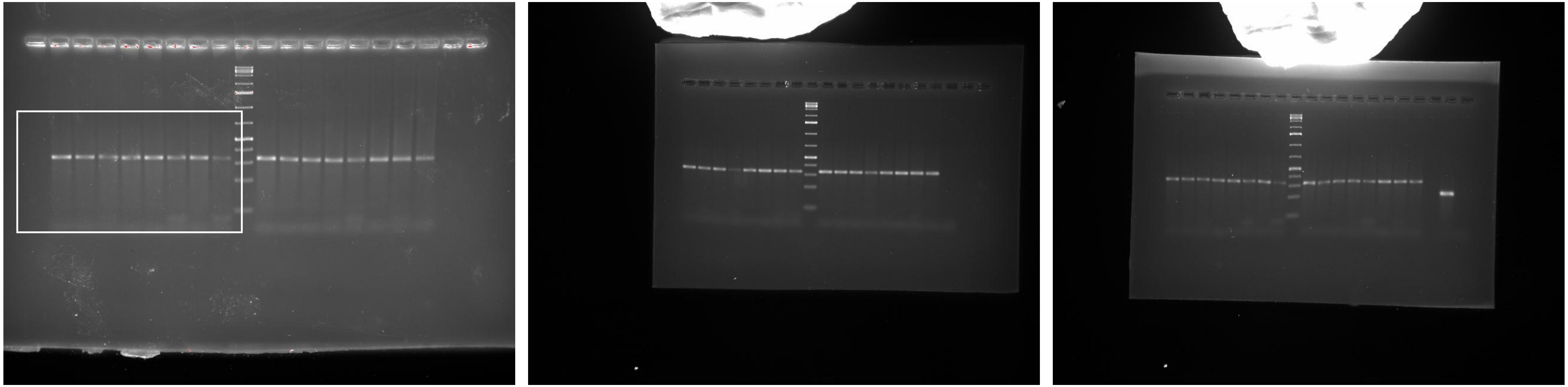

Actin of DENV

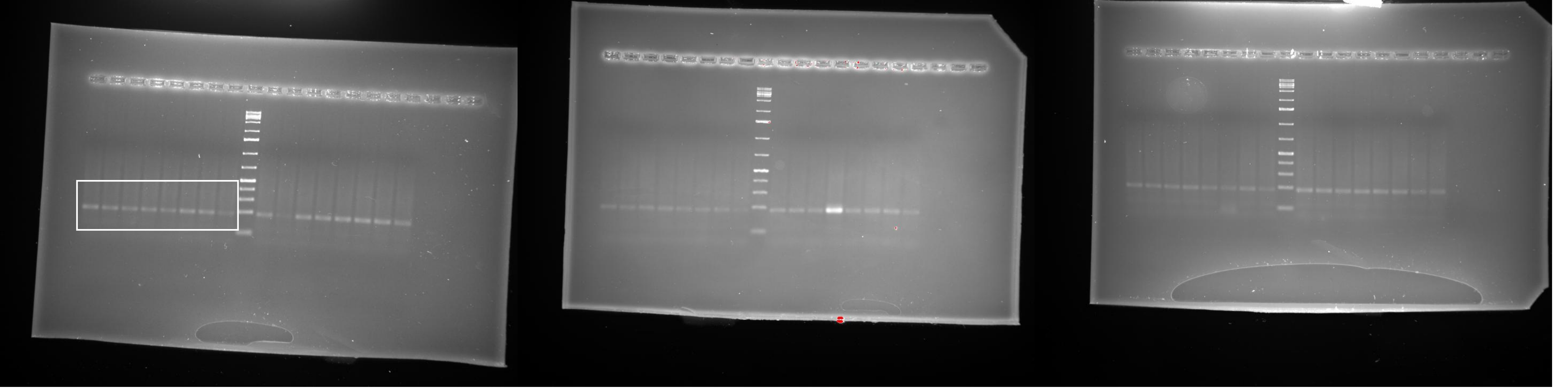

GAPDH of DENV

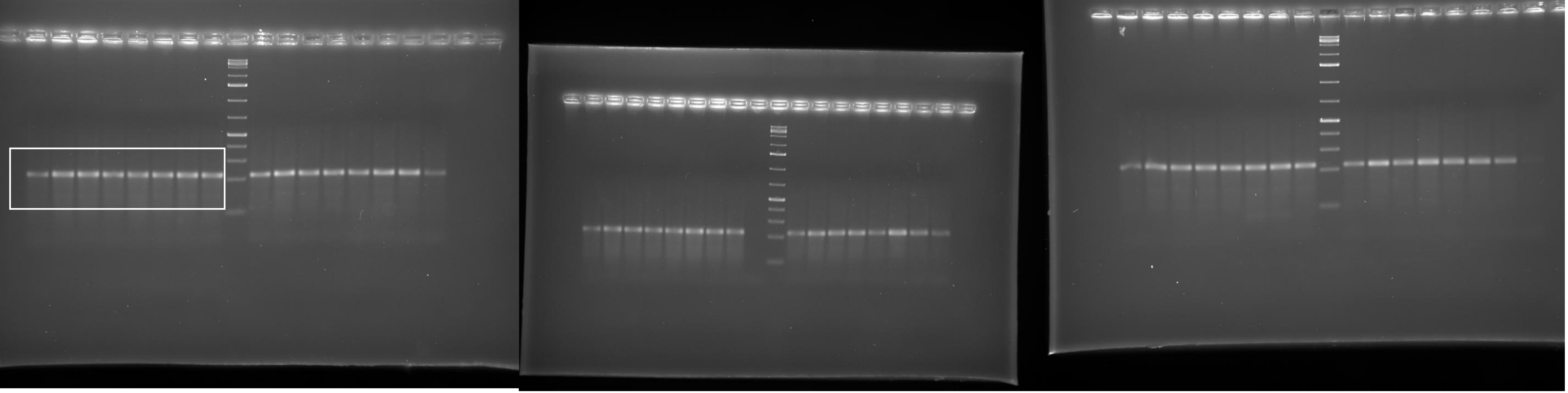

GRP78 of DENV

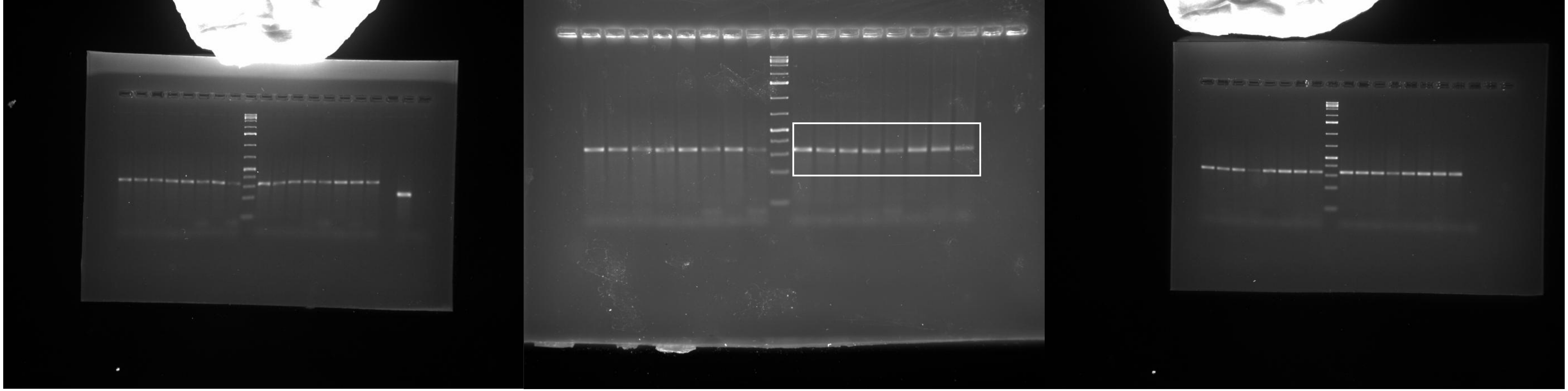

Actin of JEV

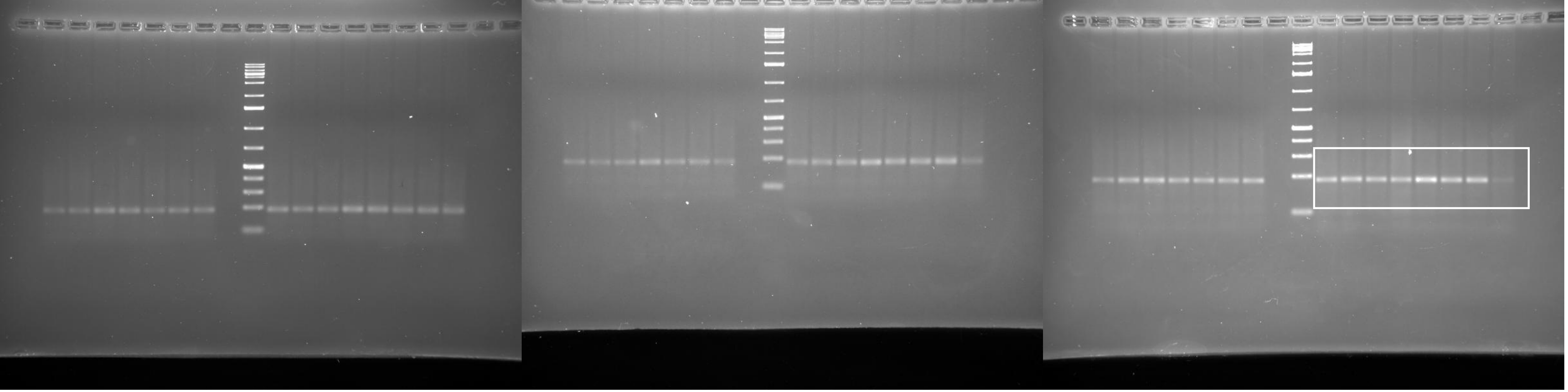

GAPDH of JEV

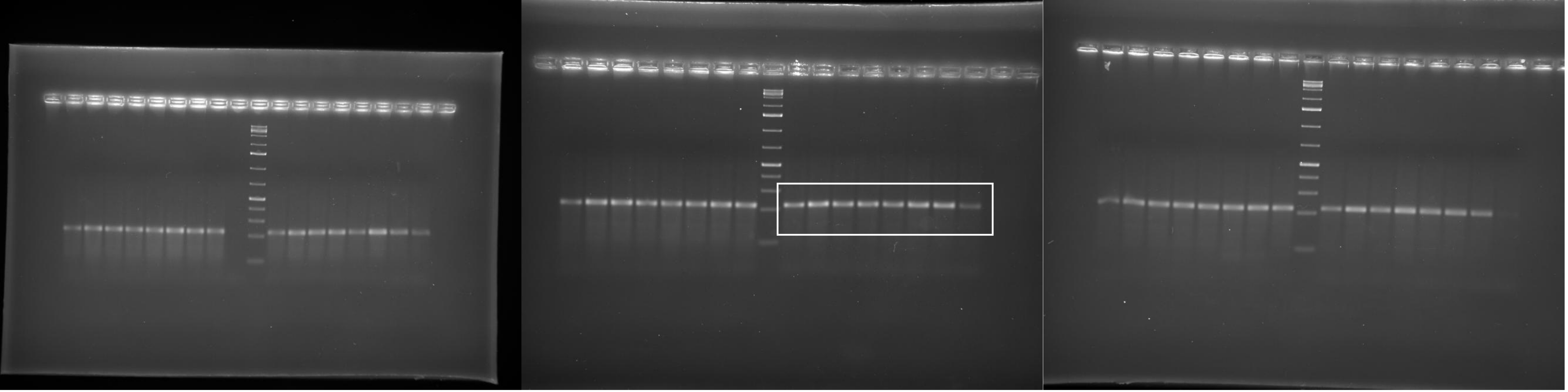

GRP78 of JEV

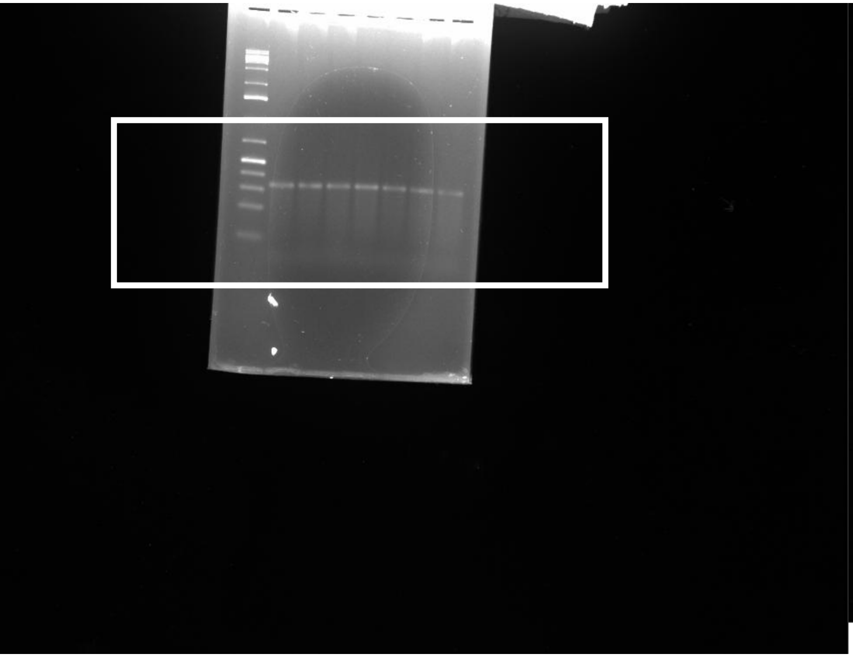
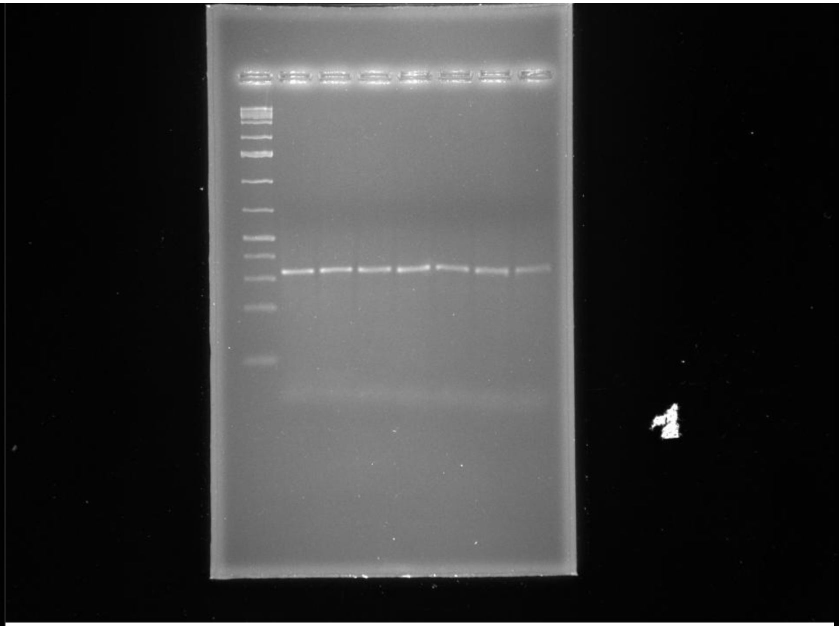
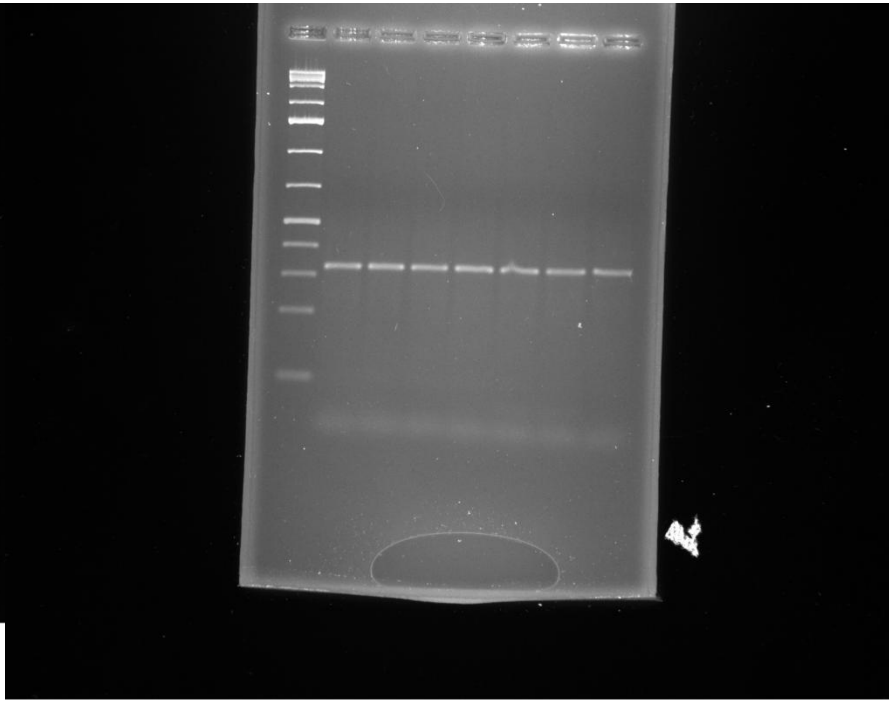

Actin control

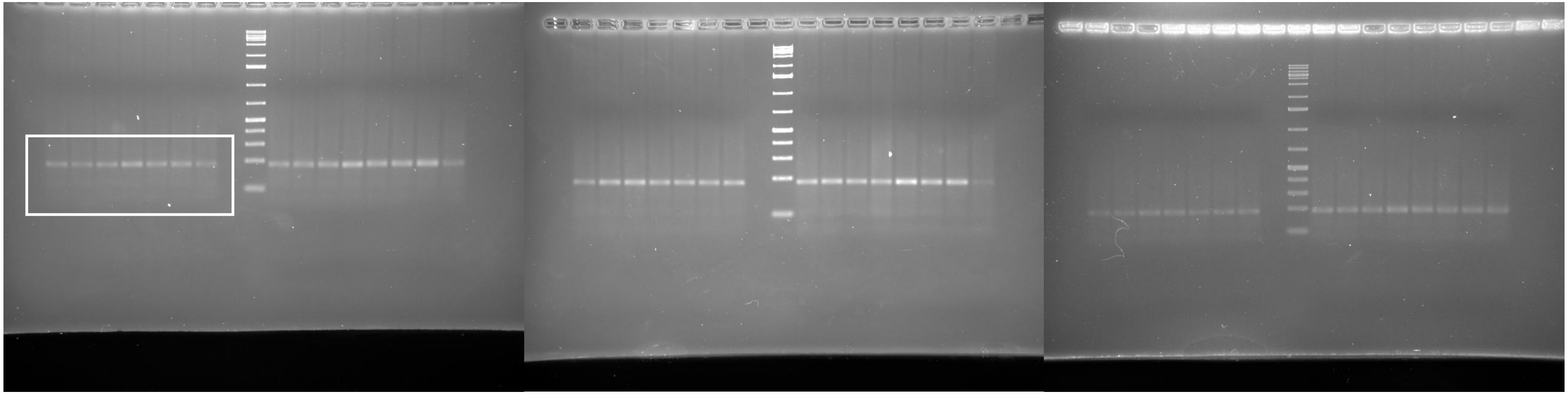

GAPDH control

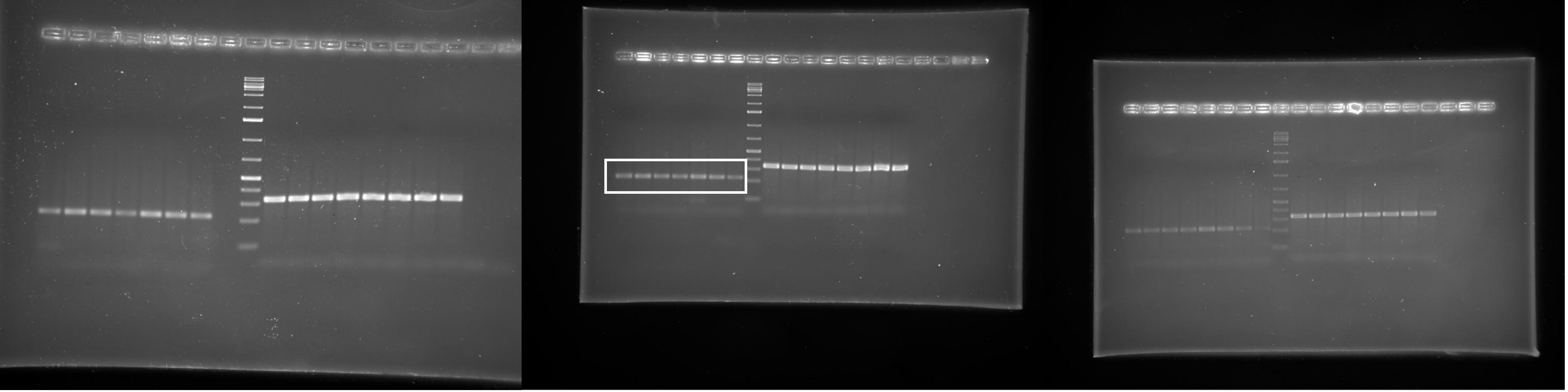

GRP78 control

GRP78 protein

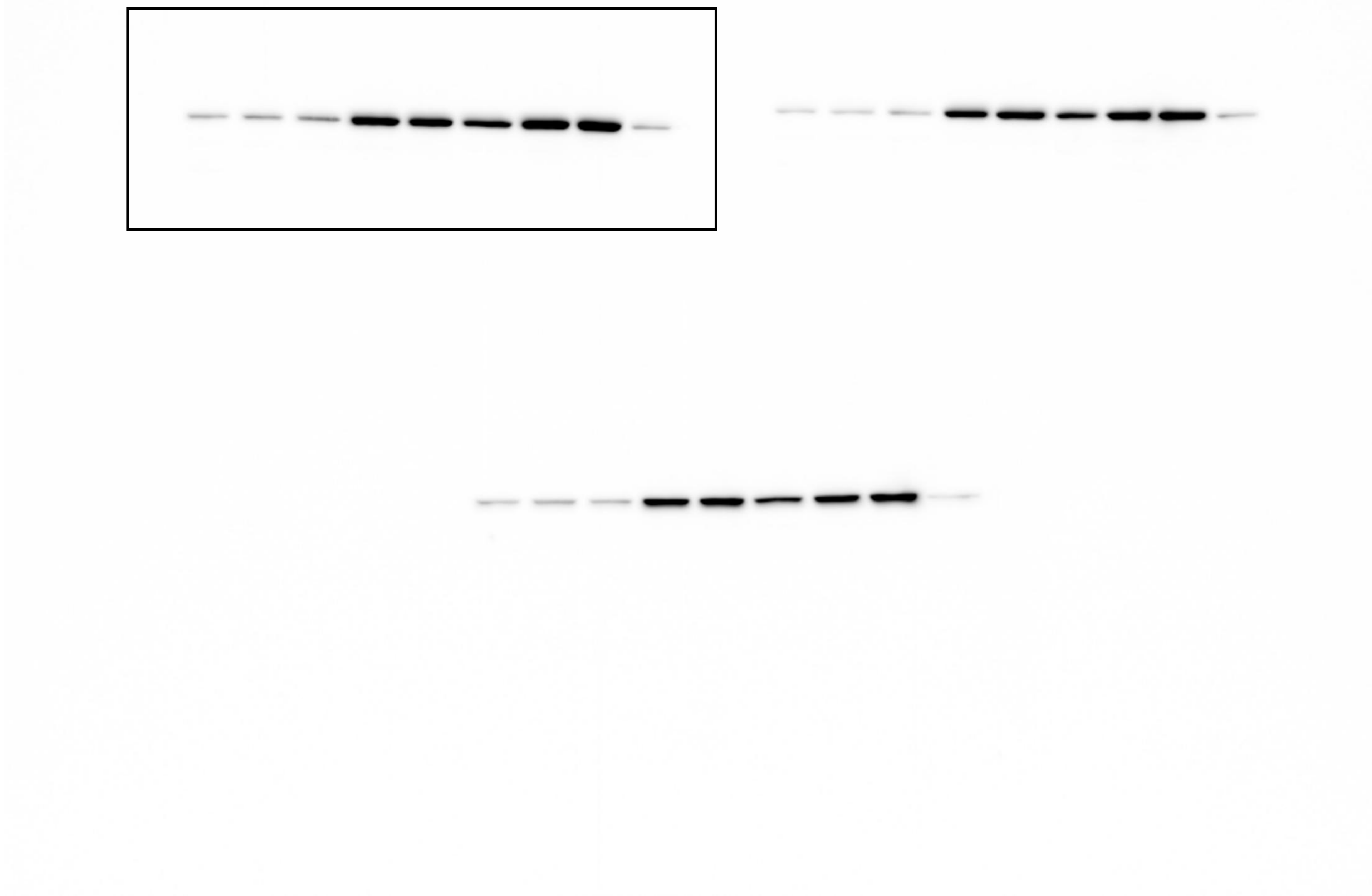

HSP70 protein

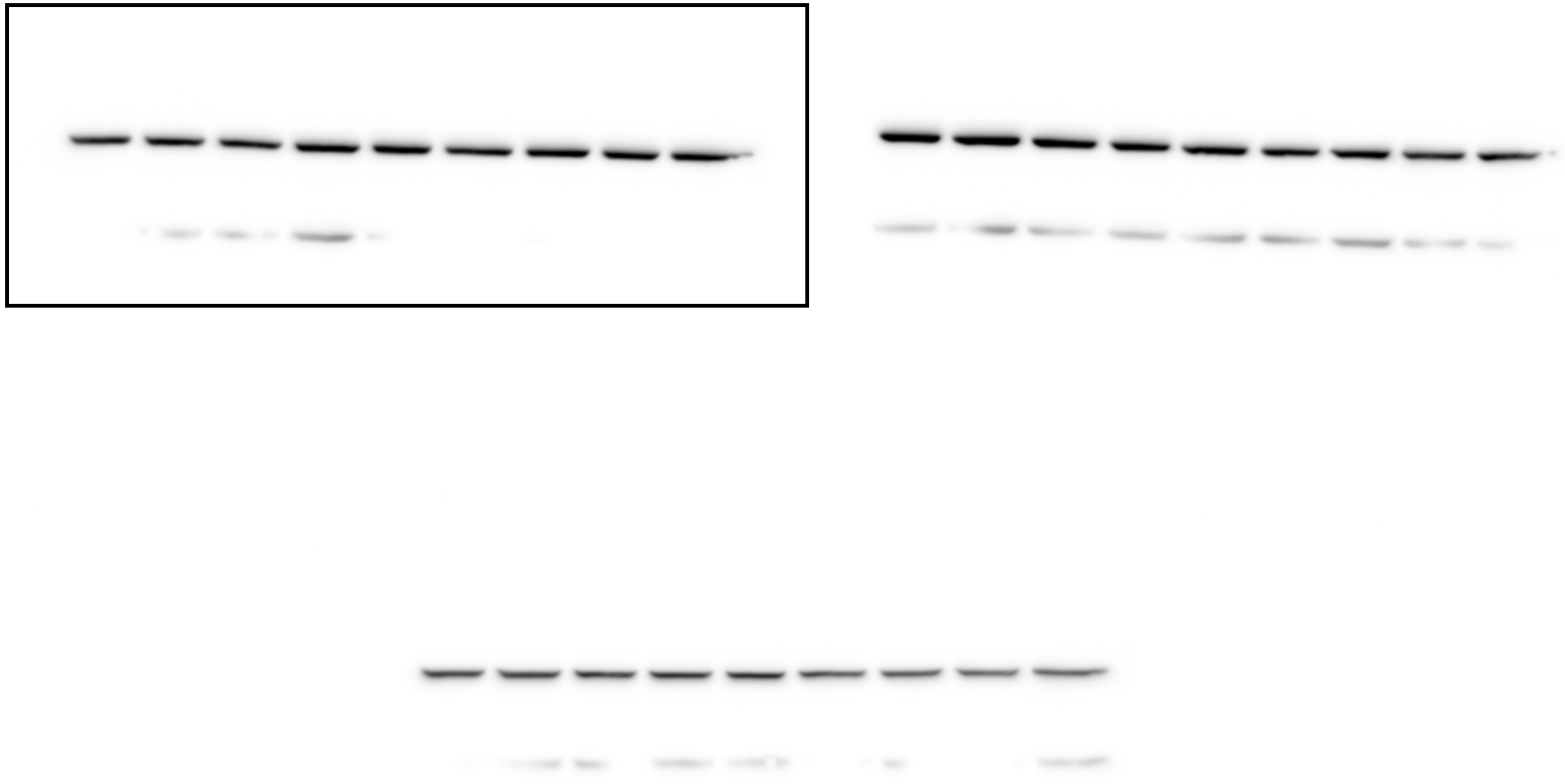

Supplement: Supplementary file 1 [file molecules-25-01246-s001.pdf]
